# Glycoside hydrolase processing of the Pel polysaccharide alters biofilm biomechanics and *Pseudomonas aeruginosa* virulence

**DOI:** 10.1038/s41522-023-00375-7

**Published:** 2023-02-02

**Authors:** Erum Razvi, Gregory B. Whitfield, Courtney Reichhardt, Julia E. Dreifus, Alexandra R. Willis, Oxana B. Gluscencova, Erin S. Gloag, Tarek S. Awad, Jacquelyn D. Rich, Daniel Passos da Silva, Whitney Bond, François Le Mauff, Donald C. Sheppard, Benjamin D. Hatton, Paul Stoodley, Aaron W. Reinke, Gabrielle L. Boulianne, Daniel J. Wozniak, Joe J. Harrison, Matthew R. Parsek, P. Lynne Howell

**Affiliations:** 1 Program in Molecular Medicine, The Hospital for Sick Children, Toronto, ON Canada; 2 Department of Biochemistry, University of Toronto, Toronto, ON Canada; 3 Department of Microbiology, University of Washington, Seattle, WA USA; 4 Department of Molecular Genetics, University of Toronto, Toronto, ON Canada; 5 Program in Developmental and Stem Cell Biology, The Hospital for Sick Children, Toronto, ON Canada; 6 Department of Microbial Infection and Immunity, The Ohio State University, Columbus, OH 43210 USA; 7 Department of Materials Science and Engineering, University of Toronto, Toronto, ON Canada; 8 Department of Biological Sciences, University of Calgary, Calgary, AB Canada; 9 Department of Microbiology and Immunology, Faculty of Medicine, McGill University, Montreal, QC Canada; 10 Infectious Disease and Immunity in Global Health, Research Institute of the McGill University Health Centre, Montreal, QC Canada; 11 McGill Interdisciplinary Initiative in Infection and Immunity, Montreal, QC Canada; 12 Department of Orthopedics, The Ohio State University, Columbus, OH 43210 USA; 13 National Biofilm Innovation Centre (NBIC) and National Centre for Advanced Tribology at Southampton (nCATS), University of Southampton, Southampton, SO17 1BJ UK; 14 Department of Microbiology, The Ohio State University, Columbus, OH 43210 USA; 15 Present Address: Département de Microbiologie, Infectiologie, et Immunologie, Faculté de Médecine Université de Montréal, Montréal, QC Canada; 16 Present Address: Department of Chemistry, Washington University, St. Louis, MO USA; 17 Present Address: Department of Biomedical Sciences and Pathobiology, VA-MD College of Veterinary Medicine, Virginia Tech, VA 24061 USA; 18 Present Address: BioVectra Inc. 11 Aviation, Charlottetown, PE Canada

**Keywords:** Biofilms, Bacteriology

## Abstract

Pel exopolysaccharide biosynthetic loci are phylogenetically widespread biofilm matrix determinants in bacteria. In *Pseudomonas aeruginosa*, Pel is crucial for cell-to-cell interactions and reducing susceptibility to antibiotic and mucolytic treatments. While genes encoding glycoside hydrolases have long been linked to biofilm exopolysaccharide biosynthesis, their physiological role in biofilm development is unclear. Here we demonstrate that the glycoside hydrolase activity of *P. aeruginosa* PelA decreases adherent biofilm biomass and is responsible for generating the low molecular weight secreted form of the Pel exopolysaccharide. We show that the generation of secreted Pel contributes to the biomechanical properties of the biofilm and decreases the virulence of *P. aeruginosa* in *Caenorhabditis elegans* and *Drosophila melanogaster*. Our results reveal that glycoside hydrolases found in exopolysaccharide biosynthetic systems can help shape the soft matter attributes of a biofilm and propose that secreted matrix components be referred to as matrix associated to better reflect their influence.

## Introduction

Biofilms are structured groups of microbial cells embedded in a self-produced matrix composed of extracellular polymeric substances (EPS) that impart emergent attributes to the community not found in planktonic cells. EPS include chemically and functionally diverse biomolecules such as nucleic acids, proteins, lipids, and exopolysaccharides. The molecular, structural, and functional roles of EPS in biofilm assembly and their physicochemical and virulence properties have collectively been termed the “matrixome”^1^. Studies of the matrixome have revealed that EPS can be grouped into two major categories based on their location within the matrix: either associated with the cell surface or secreted extracellularly. Broadly, cell-associated EPS promote adhesion, while secreted EPS support biofilm structural integrity, and protection from antibiotics and host immunity^1^. Together, both forms of EPS contribute to the biomechanics of the biofilm. In particular, their role in the formation of a diffusion-limiting barrier helps establish chemically distinct microenvironments due to gradients of pH, oxygen, signalling molecules, and other solutes across the biofilm’s three-dimensional architecture^1^. The types of EPS and the ratios of each component vary according to environmental conditions, microbial species, and strains^2^.

**Fig. 1:**
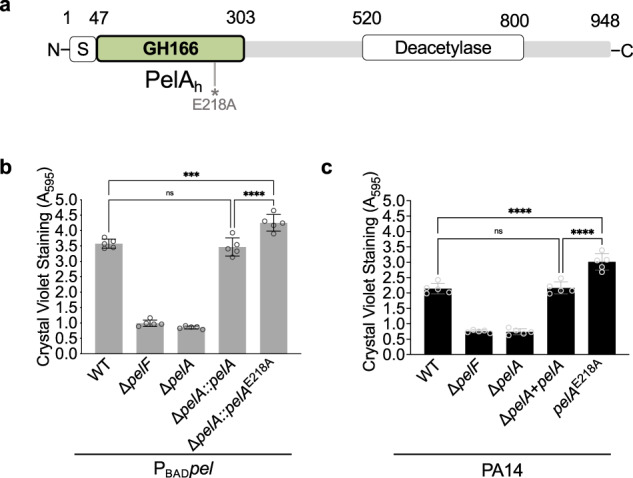
A *pelA* hydrolase mutant produces increased adherent biofilm biomass. **a** Catalytic domain architecture of PelA. S, signal sequence; GH166, glycoside hydrolase family 166 domain referred to as PelA_h_; Deacetylase; deactylase domain. * denotes the approximate location of the glycoside hydrolase catalytic variant residue, E218A. Crystal violet microtitre plate assay to quantify adherent biofilm biomass for the **b** P_BAD_ *pel* and **c** PA14 strain backgrounds, respectively. Δ*pelF* serves as a negative control. Error bars represent standard error of the mean of five independent trials. Statistical significance was evaluated using an ordinary one-way analysis of variance with Tukey corrections for multiple comparisons. ns, no significant difference, *P* ≥ 0.05; ***P < 0.001; and ****P < 0.0001. P_BAD_ *pel*, PAO1 ∆*wspF* ∆*psl* P_BAD_*pel*.

*Pseudomonas aeruginosa* is a ubiquitous Gram-negative opportunistic pathogen that is a model organism for the study of biofilms^3^. Its ability to form biofilms allows it to persist on medical devices and establish chronic urinary, respiratory, and wound infections^4^. *P. aeruginosa* is genetically capable of producing at least three exopolysaccharides; alginate, Psl, and Pel, all of which have been associated with *P. aeruginosa* lung infections in individuals with cystic fibrosis (CF)^5,6^. The Psl and Pel exopolysaccharides have also been found to play a predominant role in chronic wound infections^7,8^. In *P. aeruginosa*, the Pel polysaccharide is crucial for initiating and maintaining cell-to-cell interactions and providing protection against aminoglycoside antibiotics^9^. Pel is a positively charged, partially deacetylated α-1,4 linked *N*-acetylgalactosamine (GalNAc) polymer composed predominantly of GalNAc-GalN repeat units that exists in two forms: a high molecular weight cell-associated form and a low molecular weight secreted form^10,11^. The cationic nature of Pel enables it to bind extracellular DNA (eDNA) and host-derived anionic polymers found in CF sputum, helping to form a structural core and reduce susceptibility to antibiotic and mucolytic treatments^6,11^. In *P. aeruginosa*, all genes in the *pelABCDEFG* operon are required for Pel-dependent biofilm formation^12,13^. PelD, PelE, PelF, and PelG form a complex that provides the necessary machinery for Pel polymerization and transport across the cytoplasmic membrane^14^. PelB and PelC comprise the export machinery to guide Pel into the extracellular milieu^15,16^. PelA is a periplasmic modification enzyme with two catalytic domains: an N-terminal glycoside hydrolase domain belonging to the GH166 family and a C-terminal deacetylase domain (Fig. 1a)^17,18^.

A gene encoding a glycoside hydrolase or lyase is a common feature in many exopolysaccharide biosynthetic operons^18^. These protein products have been identified to have a variety of roles in exopolysaccharide production and biofilm formation. For example, the hydrolase activity of PslG has been demonstrated to be responsible for facilitating biofilm dispersal^19^, while the alginate lyase, AlgL, is involved in cell homeostasis, degrading aberrant polymer unable to be exported^20^. Lastly, CelC2, an endoglucanase in cellulose biosynthesis modulates the length of cellulose fibrils^21^. While mutation of catalytic residues in the PelA deacetylase domain has confirmed that its activity is required for Pel production, the role of the hydrolase domain has not been characterized^18^. Recombinant expression of the PelA hydrolase domain (residues 47-303, PelA_h_^Pa^) and its functional characterization have revealed that this domain is a retaining endo-α-1,4-*N*-acetylgalactosaminidase that can inhibit the formation of, and disrupt pre-existing, Pel-dependent biofilms when added exogenously to clinical and environmental *P. aeruginosa* isolates^17,22^. Studies have also shown that endogenous expression of *pelA* results in dispersion of *P. aeruginosa* biofilms, suggesting that the hydrolase activity of PelA is involved in matrix degradation^19^. The effect of abrogating PelA hydrolase activity specifically, and how it may be involved in Pel biosynthesis and Pel-dependent biofilm formation, remains unresolved.

In the current study, we show that loss of catalytic activity of the *P. aeruginosa* PelA hydrolase domain results in a significant increase in adherent biofilm biomass. Further, we demonstrate that the hydrolase activity of this enzyme is required for production of secreted Pel. Our data indicates that secreted Pel contributes to biofilm biomechanics by altering pellicle cohesiveness, complex colony biofilm wrinkling, matrix hydrophilicity, and stiffness. We also found that the presence of an active hydrolase enzyme reduces the virulence of *P. aeruginosa* PA14 in both *Caenorhabditis elegans* and *Drosophila melanogaster* infection models. Our results suggest that PelA hydrolase activity, in the context of Pel biosynthesis, is required for the generation of low molecular weight secreted Pel, which is important for conferring the soft matter attributes of the biofilm matrix.

## Results

### A PelA hydrolase mutant produces increased adherent biofilm biomass

We recently identified Pel biosynthetic loci in Gram-positive bacteria^23^ and have demonstrated that *Bacillus cereus* ATCC 10987 produces a Pel-like polysaccharide^24^. When the PelA hydrolase, *pelA*_*h*_^*Bc*^, was deleted in this strain we observed increased biofilm biomass, as assessed by the crystal violet microtitre dish adherence assay^24^. To determine whether this is a generalizable phenotype, and to begin to identify the role of PelA hydrolase activity in *P. aeruginosa* Pel biosynthesis, we investigated whether a comparable phenotype was observed in *P. aeruginosa*. Given that *P. aeruginosa* PelA is a bifunctional enzyme where *pelA* and its deacetylase activity are required for Pel production, we analysed the loss of hydrolase activity using an inactive catalytic point variant, E218A, that mutates the catalytic base and has been identified to abrogate PelA hydrolase activity^17,18,22,25^ (Fig. 1a). Using the crystal violet assay, we investigated biofilm formation in both a naturally Pel-producing PA14 strain background, and an engineered Pel over-producing strain background (PAO1 Δ*wspF* Δ*psl* P_BAD_*pel*, henceforth referred to herein as P_BAD_*pel*)^12,18^ (Fig. 1b, c). P_BAD_*pel* was used because, unlike PA14, high levels of *pel* transcription can be sustained and biofilm formation is not temperature dependent, allowing for facile detection of Pel polymer, Pel proteins, and Pel-dependent phenotypes^18,26^. The parental P_BAD_*pel* strain served as a positive control and formed a robust biofilm^18^. As anticipated, our negative control strain, P_BAD_*pel* Δ*pelF*, where the glycosyltransferase of the Pel system has been deleted, and our P_BAD_*pel* Δ*pelA* strain, resulted in a significant reduction of adhesion (Fig. 1b). Complementation of P_BAD_*pel* Δ*pelA* with a wild-type copy of *pelA* restored biofilm formation, comparable to the parental P_BAD_*pel* strain (Fig. 1b). Interestingly, P_BAD_*pel* Δ*pelA* complemented with a *pelA* hydrolase catalytic point variant, P_BAD_*pel* Δ*pelA*::*pelA*^E218A^, resulted in a significant increase in adherent biofilm biomass relative to P_BAD_*pel* and P_BAD_*pel* Δ*pelA*::*pelA* (Fig. 1b). The phenotype observed is not linked to the overexpression of Pel as a PA14 PelA hydrolase mutant strain, PA14 *pelA*^E218A^, where the catalytically inactive point variant allele is at the native locus, displayed a significant increase in adherent biofilm biomass relative to both PA14 and PA14 Δ*pelA* + *pelA* (Fig. 1c). Our PA14 Δ*pelF* negative control and PA14 Δ*pelA* strains, as anticipated, exhibited a significant reduction of adhesion (Fig. 1c). The crystal violet assays reveal that abolishing PelA hydrolase activity in P_BAD_*pel* and PA14, as well as *B. cereus* ATCC 10987^24^, leads to an increase in adherent biofilm biomass, which may be a general phenomenon when hydrolase activity in a Pel biosynthetic system is nullified.

### Cells lacking PelA hydrolase activity exhibit wild-type viable cell numbers and morphology

**Fig. 2:**
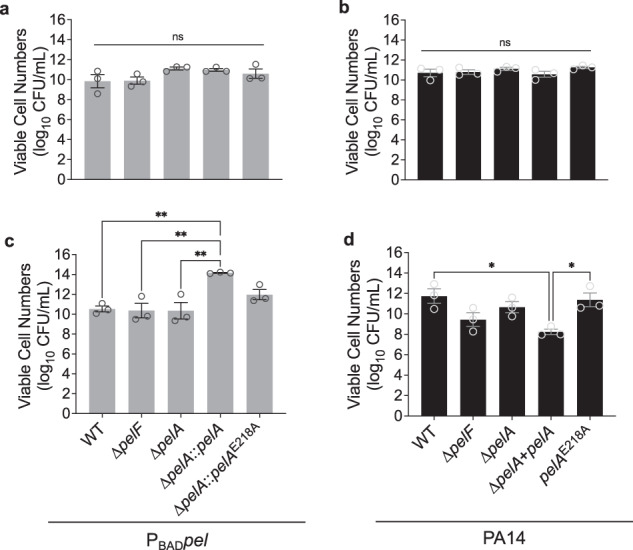
CFU counts from P_BAD_*pel* and PA14 strains show no compromise in viable cell numbers. CFU counts from planktonic cultures of indicated **a** P_BAD_ *pel* and **b** PA14 strains, respectively. Error bars represent standard error of the mean of three independent trials. CFU counts from biofilms for **c** P_BAD_ *pel* and **d** PA14 strains, respectively. Error bars represent standard error of the mean of three independent trials. Statistical significance was evaluated using an ordinary one-way analysis of variance with Tukey corrections for multiple comparisons. ns no significant difference, *P* ≥ 0.05; and ***P < 0.0001. P_BAD_ *pel*, PAO1 ∆*wspF* ∆*psl* P_BAD_*pel*.

Deletion of alginate lyase (*algL*) in strains that overproduce alginate can compromise viable cell numbers^20,27^. Thus, we used transmission electron microscopy (TEM) to examine the cellular morphology of our strains. All strains were rod-shaped with a preserved cell envelope and a taut outer membrane, which are normal *P. aeruginosa* cell morphologies^28^ (Supplementary Fig. 1). The apparent increase in adherent biofilm biomass in the PelA^E218A^ hydrolase variant strains could be due to attenuated viability at later assay time points, where increased cell death could equate to more biofilm biomass deposition. Therefore, we also assessed viable cell numbers by enumerating colony-forming units (CFUs) from planktonic cultures. All strains showed no significant differences in viable cell numbers (Fig. 2a, b). We also enumerated CFU counts from biofilms grown in a 96-well plate and disrupted with exogenously added PelA_h_^Pa^ to release cells (Fig. 2c, d). For the P_BAD_*pel* Δ*pelA*::*pelA* strain, a significant increase in CFU/mL was observed relative to the other P_BAD_*pel* strains (Fig. 2c), but not when grown planktonically (Fig. 2a). Similarly, for the PA14 Δ*pelA* + *pelA* complemented strain, a modestly significant change, in this case, a decrease, in CFU/mL was observed (Fig. 2d). It is unclear why there are significant differences in viable cell numbers when the P_BAD_*pel* Δ*pelA* and PA14 Δ*pelA* strains are complemented with wild-type *pelA*. We speculate that this may be due to differences in the expression levels of PelA in both strain backgrounds that alter the length of the Pel exopolysaccharide secreted, thereby influencing the efficiency with which PelA_h_^Pa^ can release biofilm cells for enumeration. No significant differences in CFUs were observed for any of the other P_BAD_*pel* and PA14 strains. Taken together, these data confirm that the increase in adherent biofilm biomass relative to wild-type in a PelA hydrolase point mutant strain is not the result of compromised viable cell numbers or morphology. This suggests that PelA hydrolase activity plays a role in biofilm adherence.

### The hydrolase activity of PelA generates secreted Pel

**Fig. 3:**
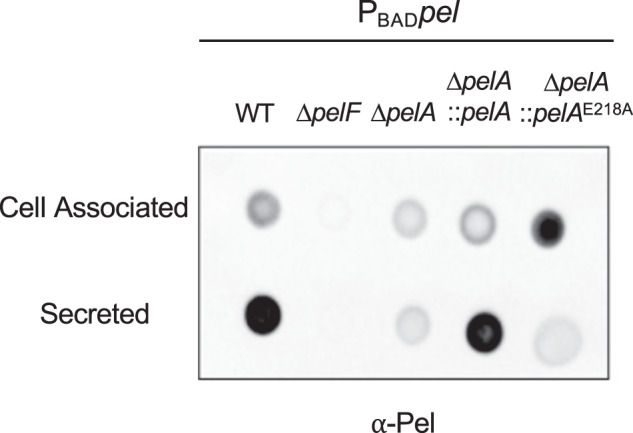
PelA hydrolase activity is responsible for generating secreted Pel. Dot blot of crude cell-associated and secreted Pel samples from the indicated strains. Pel was detected using α-Pel primary antibody with HRP-conjugated secondary antibody. P_BAD_ *pel*, PAO1 ∆*wspF* ∆*psl* P_BAD_*pel*.

The Pel exopolysaccharide has previously been shown to exist in two different forms, a high molecular weight cell-associated form and low molecular weight secreted form^11^. How the two forms of Pel are synthesized is currently unknown. As cell-associated matrix components are often involved in adherence^1^, we reasoned that the increased crystal violet staining observed for the PelA hydrolase mutants could be due to changes in levels of secreted Pel. To test this hypothesis, crude cell-associated and secreted fractions of Pel from the P_BAD_*pel* strains were analyzed using a dot blot and Pel-specific polyclonal antibody (α-Pel;^18^). The PA14 strain is not amenable to analysis with α-Pel due to cross-reactivity between the antiserum and the PA14 O-specific antigen^18^. As anticipated, a robust signal was observed for cell-associated and secreted Pel from the wild-type P_BAD_*pel* strain (Fig. 3). No signal was observed in either fraction from the P_BAD_*pel* Δ*pelF* strain. Unexpectedly, the P_BAD_*pel* Δ*pelA* strain showed a weak signal for both the cell-associated and secreted Pel fractions. Whole genome sequencing of P_BAD_*pel* Δ*pelA* confirmed that this strain is isogenic to P_BAD_*pel* and therefore this result is strictly due to the mutation in *pelA*. We hypothesize that sparse amounts of acetylated Pel are being released *via* cell lysis during handling because this strain does not form a Pel-dependent biofilm (Fig. 1b). A comparable hypothesis was proposed for Δ*pgaB* in the poly-β-1,6-*N*-acetyl-D-glucosamine (PNAG) exopolysaccharide system^29^. PgaB, like PelA, is an enzyme with both glycoside hydrolase and deacetylase activity^30^. Analysis of the complemented P_BAD_*pel* Δ*pelA*::*pelA* strain revealed a signal for cell-associated and secreted Pel comparable to the P_BAD_*pel* parental strain. Interestingly, when the activity of the hydrolase was compromised in the P_BAD_*pel* Δ*pelA*::*pelA*^E218A^ strain, there was a robust signal for cell-associated Pel, while the secreted Pel signal was comparable to that of the P_BAD_*pel* Δ*pelA* strain (Fig. 3). Collectively, these results suggest that the hydrolase activity of PelA generates the secreted form of the Pel polymer.

### Cell-associated Pel is a key component of flow cell biofilm microcolonies

**Fig. 4:**
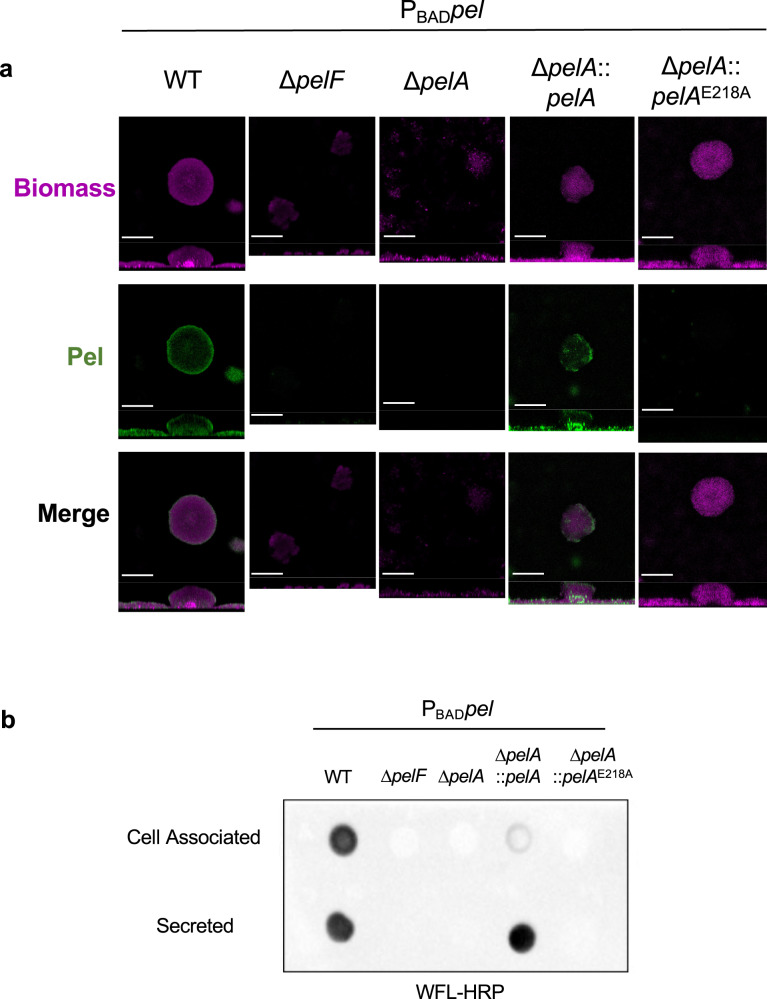
Cell-associated Pel is a key component of biofilm microcolonies in flow cells. **a** Representative confocal flow cell images stained with SYTO62 (pink) and the Pel-specific lectin WFL-FITC (green) to detect biomass and Pel, respectively. Scale bars = 100 *μ*m. **b** Dot blot of crude cell-associated and secreted Pel fractions of the indicated strains. Pel was detected using WFL-HRP. P_BAD_ *pel*, PAO1 ∆*wspF* ∆*psl* P_BAD_*pel*.

Pel is localized to the stalk and periphery of flow cell biofilms^11^. To determine whether secreted Pel plays a role in biofilm architecture, we next examined the morphology of Pel-dependent flow cell biofilms using confocal microscopy. As previously demonstrated^11^, fluorescein-labelled *Wisteria floribunda* lectin (WFL-FITC) staining, which recognizes terminal GalNAc moieties, detected Pel at the biofilm stalk and periphery in the P_BAD_*pel* strain (Fig. 4a)^11,31^. No staining was detected in the P_BAD_*pel* Δ*pelF* and P_BAD_*pel* Δ*pelA* strains (Fig. 4a). The complemented P_BAD_*pel* Δ*pelA*::*pelA* strain, which produces cell-associated and secreted Pel (Fig. 3), exhibited WFL-FITC signal localized to the biofilm stalk and periphery similar to wild type P_BAD_*pel*. To our surprise, there was no WFL-FITC signal in a P_BAD_*pel* Δ*pelA*::*pelA*^E218A^ flow cell biofilm despite it forming a microcolony similar to wild-type. To further examine this, we performed a dot blot using WFL conjugated to horseradish peroxidase (WFL-HRP) with crude cell-associated and secreted Pel fractions (Fig. 4b). The WFL-HRP dot blot detected Pel in the cell-associated and secreted fractions of P_BAD_*pel* but not the P_BAD_*pel* Δ*pelF* and P_BAD_*pel* Δ*pelA* negative controls. In addition, while a comparable signal relative to P_BAD_*pel* was observed for the secreted fraction in the P_BAD_*pel* Δ*pelA*::*pelA* strain, a poor signal was observed for the cell-associated Pel fraction. A Western blot probing for PelA expression levels shows that P_BAD_*pel* Δ*pelA*::*pelA* has increased PelA expression relative to the parental P_BAD_*pel* strain, suggesting that increased PelA hydrolase activity may result in the generation of less cell-associated Pel (Supplementary Fig. 2). Interestingly, unlike the α-Pel dot blot, no signal was detected in the P_BAD_*pel* Δ*pelA*::*pelA*^E218A^ strain for either cell-associated or secreted Pel fractions (Fig. 4b). Taken together, our microscopy of the P_BAD_*pel* strains suggest that PelA hydrolase activity is dispensable for microcolony formation in a flow cell. The ability of α-Pel (Fig. 3) and inability of WFL to detect cell-associated Pel in the P_BAD_*pel* Δ*pelA*::*pelA*^E218A^ strain may have implications for the length of the polymer, as the lack of hydrolase activity likely results in longer polymers with fewer terminal GalNAc moieties for WFL to recognize. This hypothesis is reinforced by the heavy flocculation consistently observed in overnight cultures of the P_BAD_*pel* Δ*pelA*::*pelA*^E218A^ and PA14 *pelA*^E218A^ hydrolase mutants (Supplementary Fig. 3), which may be indicative of increased polymer bridging, a mechanism of aggregation that occurs when polymers simultaneously adsorb on cells and bring them together, and has been observed in P. aeruginosa aggregates in CF sputum^6,32,33^. Thus, qualitatively, our results suggest that cell-associated rather than secreted Pel plays the dominant role in forming flow cell biofilm microcolonies.

### Secreted Pel promotes pellicle buoyancy and cohesiveness

**Fig. 5:**
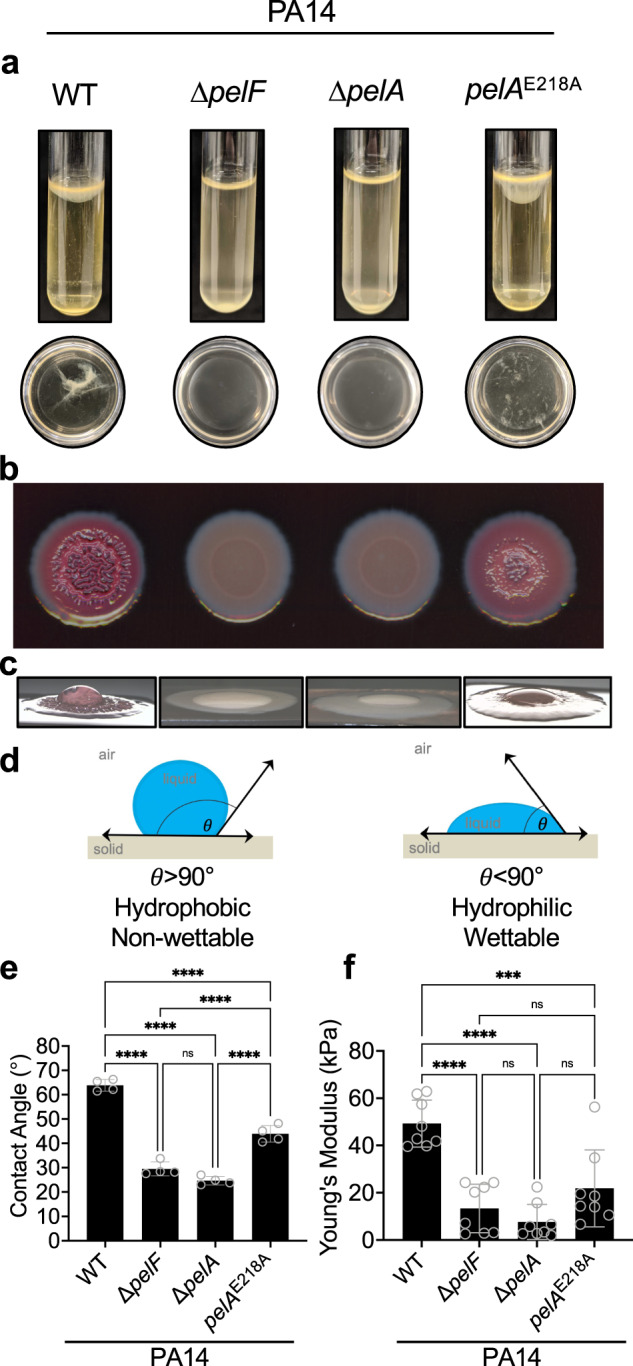
Secreted Pel contributes to the physical and mechanical properties of PA14 biofilms. **a** Standing pellicle assay. Top, pellicles of the indicated strains grown in borosilicate glass tubes. Bottom, corresponding pellicles poured into a petri dish. **b** Congo red colony morphologies. **c** Representative images of water droplets on colony biofilms used for the contact angle measurements. **d** Illustration of contact angle (*θ*) measurement and its implication for surface hydrophobicity. **e** Contact angle hydrophobicity assay. Error bars represent standard deviation of four independent trials. **f** Young’s modulus quantified from uniaxial indentation rheology. Error bars represent standard deviation of eight independent trials. Statistical significance was evaluated using an ordinary one-way analysis of variance with Tukey corrections for multiple comparisons. ns, no significant difference, *P* ≥ 0.05; ***P < 0.001, ****P < 0.0001.

Given that the morphologies of the P_BAD_*pel* and P_BAD_*pel* Δ*pelA*::*pelA*^E218A^ flow cell biofilms were similar, we next sought to determine whether the secreted Pel produced by the hydrolase activity of PelA has a functional role in the biofilm matrix. To examine this, we used the PA14 strain background, as it naturally produces Pel as the dominant matrix exopolysaccharide^12^. Pseudomonads can produce pellicles, or biofilms at an air-liquid interface, in static liquid cultures with varying morphology, strength, adherence, and physical integrity^34–36^. As Pel is known to interact with other matrix components^6,11,37^, we hypothesized that lack of secreted Pel may influence pellicle morphology. To investigate this, we grew the PA14 strains in static cultures to allow for pellicle formation, and as has been established previously, examined the physical integrity of matrix material ex situ by tipping the culture into a petri dish^34–36^ (Fig. 5a). As expected, PA14 formed a buoyant pellicle at the air-liquid interface that was visibly attached to the vial walls. When tipped into a petri dish, the pellicle retained its physical integrity as a flexible single structure. Consistent with our crystal violet assays, PA14 Δ*pelF* and PA14 Δ*pelA* did not form pellicles^12,18^, while PA14 *pelA*^E218A^ formed a pellicle that visibly sagged more than wild-type PA14, indicating poorer buoyancy. Tipping the contents of the PA14 *pelA*^E218A^ tube into a petri dish revealed that the pellicle had poor cohesion and formed multiple flocs. These observations suggest that the PA14 *pelA*^E218A^ pellicle is more fragile than the wild-type pellicle and that the generation of secreted Pel enhances pellicle buoyancy and cohesiveness.

### Production of secreted Pel is required for increased wrinkling of colony biofilms

PA14 can also form colony biofilms that have architecturally complex wrinkling patterns. Wrinkle extrusion increases the surface area to volume ratio as a strategy to promote access to O_2_, thus balancing the intracellular redox state of resident biofilm cells^38,39^. Pel is required for the pronounced central and spoke wrinkles observed in PA14 colony biofilms^38^. Since secreted Pel contributes to the buoyancy and cohesiveness of pellicles, we next used a Congo red colony morphology assay to examine wrinkling and to determine if secreted Pel plays a role in colony biofilm morphological development. As anticipated, we observed robust wrinkle formation and Congo red adsorption in PA14, and smooth pale pink colonies in our PA14 Δ*pelF* and PA14 Δ*pelA* negative controls (Fig. 5b)^12^. Strikingly, PA14 *pelA*^E218A^ formed a colony biofilm with only a thick central wrinkle. The spoke wrinkles were absent and replaced with a smooth surface lacking vertical structures (Fig. 5b). Examining the morphology of the colony biofilms up to nine days indicated that the absence of spoke wrinkles in PA14 *pelA*^E218A^ was not due to delayed morphological development (Supplementary Fig. 4). This data reveals that the generation of secreted Pel plays a role in PA14 colony biofilm wrinkle formation.

### PelA hydrolase activity decreases the wettability of P. aeruginosa biofilms

The alterations observed in PA14 *pelA*^E218A^ colony biofilm morphology are reminiscent of the colony biofilms formed by *Bacillus subtilis* when the amphiphilic secreted biofilm matrix protein, BslA, is deleted^40,41^. BslA is a major contributor to the hydrophobic surface layer of *B. subtilis* colony biofilms. Surface chemistry and wrinkling work in tandem to give a colony biofilm hydrophobic characteristics^42^. Wrinkles provide texture and allow for the formation of microscopic cavities that trap air underneath a liquid droplet, thus conferring surface hydrophobicity^40^. Given the colony morphologies observed, we hypothesized that a PA14 *pelA*^E218A^ colony biofilm could have altered wetting properties relative to wild-type PA14. To probe this, we performed a colony surface hydrophobicity assay and examined the wetting properties of the biofilm by measuring the contact angle of a droplet of water on the colony surface^41,42^. Contact angles serve as a quantitative measure of wettability, where an angle >90° indicates non wettable/hydrophobic properties, and an angle <90° indicates wettable/hydrophilic properties^42^ (Fig. 5d). The PA14 strains all have contact angles less than 90° indicating that the surfaces are all relatively hydrophilic (Fig. 5c, e). Our data show that the mutants, PA14 Δ*pelF*, PA14 Δ*pelA*, and PA14 *pelA*^E218A^ are all more hydrophilic than the wild-type PA14 strain with significantly lower average contact angles. Interestingly, the contact angle of PA14 *pelA*^E218A^ was also significantly higher relative to PA14 Δ*pelF* and PA14 Δ*pelA* (Fig. 5e). Together, this indicates that Pel production generally decreases wettability and that the production of secreted Pel is required to maximize this effect.

### PelA hydrolase activity increases the stiffness of biofilms

To determine if secreted Pel impacts the mechanical properties of a colony biofilm, we next performed indentation rheology to examine stiffness or elasticity, which is a corollary of biofilm structure and composition^43^. Uniaxial indentation, where biofilms are compressed and the force required is measured, was performed on 7-day PA14 colony biofilms. From this analysis, the Young’s modulus, which is a measure of the stiffness, or the extent that biofilms can resist compression under a normal force, was quantified for each strain^44,45^. This revealed that loss of either *pelF* or *pelA*, resulted in significantly reduced Young’s modulus, compared to wild-type PA14 biofilms (Fig. 5f). Similarly, PA14 *pelA*^E218A^ resulted in a significantly reduced Young’s modulus compared to wild-type PA14 biofilms. Furthermore, the Young’s modulus of PA14 *pelA*^E218A^ biofilms were not statistically different from those of PA14 Δ*pelF* and PA14 Δ*pelA* biofilms (Fig. 5f). This indicates that secreted Pel specifically contributes to the development of stiffer biofilms, conferring mechanical properties that are lacking in strains that only produce cell associated Pel.

### PelA hydrolase activity reduces P. aeruginosa PA14 virulence

**Fig. 6:**
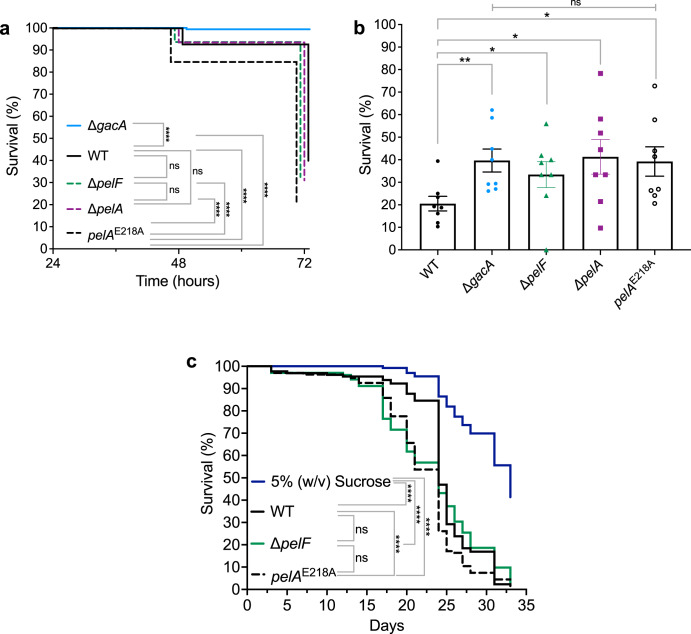
PelA hydrolase activity reduces *P. aeruginosa* PA14 virulence. **a** *C. elegans* slow killing assay Kaplan-Meier survival curve for PA14 strains. Data are pooled from three independent experiments (*n* = 105–120 worms per PA14 strain per experiment). Statistical significance was evaluated using a log-rank Mantel-Cox test. **b** *C. elegans* fast killing for PA14 strains. Survival was assessed after 24 h. Error bars represent standard error of the mean of eight independent experiments (*n* = 35–40 worms per PA14 strain per experiment). Statistical significance was evaluated using a one-tailed, unpaired Student’s *t*-test with Bonferroni corrections for multiple testing. **c** *D. melanogaster* oral infection Kaplan-Meier survival curve for PA14 strains. Data are pooled from six independent experiments (*n* = 20 flies per PA14 strain per replicate). Survival was assessed every 24 hours. Statistical significance was evaluated using a log-rank Mantel-Cox test. ns, no significant difference, *P* ≥ 0.05; *P = 0.01–0.05; **P = 0.001–0.01; and ****P < 0.0001.

The viscoelasticity of *P. aeruginosa* biofilms has been proposed to be a virulence property^43,45^. Given the results of our rheology experiments, we next sought to determine if the virulence of PA14 *pelA*^E218A^ was different from that of the wild-type strain. Though we have no direct evidence that PA14 *pelA*^E218A^ has higher levels of cell-associated Pel, our data in Fig. 5 provides indirect evidence as the hydrolase mutant strain consistently differs from wild type. First, we examined PA14 pathogenesis in *C. elegans* to determine host survival using two established agar-based assays that differ in the PA14 virulence factors responsible for killing^46,47^. The PA14 Δ*gacA* strain served as an avirulent negative control, as it fails to accumulate in the *C. elegans* intestinal lumen. Deletion of the two-component response regulator *gacA* also prevents the synthesis of small toxins such as cyanide and pyocyanin^47^. PA14 Δ*pelF* and Δ*pelA* served as non-biofilm, non-Pel producing controls. We first performed a slow-killing assay, where nematode death is due to active infection of the gut *via* consumption of PA14 grown on low osmolarity minimal media^46,47^. As expected, infection with the PA14 Δ*gacA* strain resulted in minimal death (Fig. 6a). In contrast, relative to PA14 Δ*gacA*, wild-type PA14 had a significantly increased rate of killing with ~40% *C. elegans* survival after 72 h. Interestingly, a log-rank Mantel-Cox test to evaluate the differences in killing kinetics revealed that PA14 *pelA*^E218A^ had a significantly increased rate of killing relative to the wild-type PA14, Δ*gacA*, Δ*pelF*, and Δ*pelA* strains with only ~20% *C. elegans* survival after 72 h, suggesting that the presence of only cell associated Pel increases host killing kinetics (Fig. 6a). To determine if death is caused by diffusible toxins and not live bacteria, we also performed a fast killing assay, where nematode death is due to secreted PA14 toxin production *via* consumption of PA14 grown on high osmolarity minimal media (Fig. 6b)^46,47^. In this assay we found no significant difference in killing between PA14 Δ*gacA*, Δ*pelF*, Δ*pelA*, and *pelA*^E218A^ which suggests that toxin production may be compromised in these strains (Fig. 6b). Wild-type PA14 resulted in significantly more nematode killing *via* toxin production relative to PA14 Δ*gacA*, Δ*pelF*, Δ*pelA*, and *pelA*^E218A^ (Fig. 6b). The PelA hydrolase point mutant strain, PA14 *pelA*^E218A^, in a slow killing assay had a significant increase in killing relative to wild type, and in a fast killing assay a significant reduction in killing relative to wild type (Fig. 6a,b). Together, these results reveal that when PA14 *pelA*^E218A^ actively colonizes the nematode gut, it kills a *C. elegans* host at a faster rate than wild-type PA14. In addition, hydrolase activity appears to be necessary for increased killing of *C. elegans* by toxin production.

To corroborate the results in the *C. elegans* survival assays, we next examined host survival in an established *D. melanogaster* oral infection model developed to study *P. aeruginosa* biofilm infections in vivo. The advantage of this model is that the innate immune system of *Drosophila* has similarities with the vertebrate innate immune system, thus aiding our understanding the role PelA’s hydrolase activity may play in virulence in mammals^48^. As a negative control, *Drosophila* were fed 5% (w/v) sucrose. As observed in the *C. elegans* slow-killing assay, PA14 *pelA*^E218A^ had a significantly increased rate of *Drosophila* killing relative to wild-type PA14 (Fig. 6c), but not Δ*pelF*. This indicates that that the biofilms formed by wild type and PA14 *pelA*^E218A^ are not the same and suggests that loss of PelA hydrolase activity increases host-killing kinetics in both infection models. Collectively, we observed a consistent increased rate of host killing by PA14 *pelA*^E218A^ suggesting that PelA’s hydrolase activity plays a role in reducing virulence.

## Discussion

**Fig. 7:**
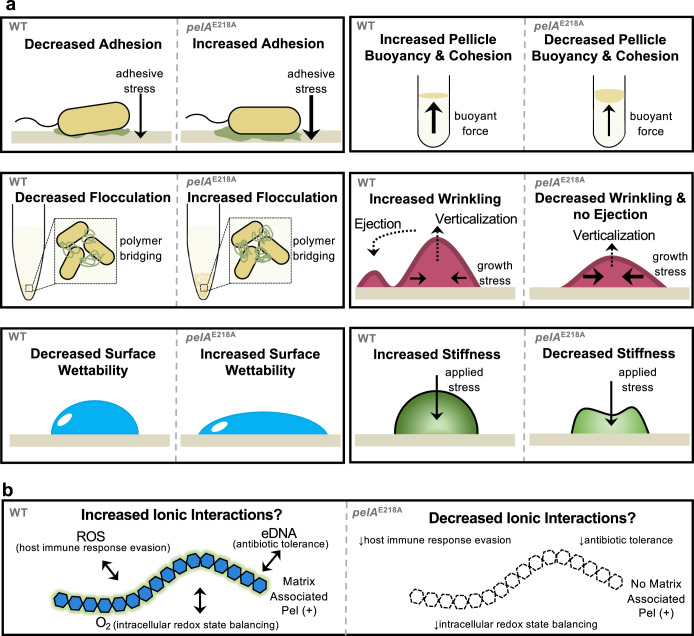
Summary of results and implications. Schematic of **a** Properties that PelA hydrolase activity imparts to *P. aeruginosa* PA14 Pel biofilm biomechanics. **b** Additional properties implicated by our study. ROS reactive oxygen species.

The role of PelA hydrolase activity in the context of Pel biosynthesis has not previously been explored. Herein, we show that PelA processes Pel to generate the low molecular weight secreted form of the polymer and that the presence of secreted Pel in the biofilm matrix reduces the adherence of cells. Notably, we discovered that PelA hydrolase activity confers beneficial biomechanical properties to Pel biofilms and decreases *P. aeruginosa* virulence (Fig. 7).

Cell-to-surface adhesion influences overall biofilm morphology, but the underlying mechanisms of how biofilm matrix components influence biofilm architecture are poorly understood^49,50^. In our studies, we found that abrogation of PelA’s hydrolase activity led to increased adherence (Fig. 1b, c) and altered phenotypes in multiple biofilm models. The morphology of *P. aeruginosa* aggregates in CF sputum was previously found to be consistent with a polymer bridging mechanism that brings cells together through polymer and cell interactions^6,32,33^. The heavier flocculation of the PelA hydrolase mutant relative to the wild-type strain (Supplementary Fig. 3) suggests that hydrolase activity may function to reduce polymer bridging and that the higher molecular weight cell-associated form of Pel plays a role in this type of aggregation^6,32,33^.

Most Pseudomonads form pellicles under static growth conditions as this promotes access to O_2_^34,39^. Mechanisms that enable pellicle formation include the synthesis of a matrix that prevents mixing with liquid media, trapping of gas accumulated through metabolite processing to confer buoyancy, and/or adherence to the walls of a container^51^. Visualized in a glass tube, and ex situ in a petri dish, the PA14 *pelA*^E218A^ pellicle was less buoyant and cohesive than wild-type (Fig. 5a). Our data also shows that the hydrolase mutant adheres better to a solid surface (Fig. 1) and exhibits heavier flocculation (Supplementary Fig. 3). This suggests that interactions with other matrix components known to interact with Pel, such as eDNA and the extracellular adhesin CdrA, may be compromised when secreted Pel is absent^6,11,37^. Loss of matrix interactions could result in a less cohesive pellicle and thus mixing with media and/or release of gas. Collectively this would lead to a less buoyant pellicle. As oxygenation influences the chemistry and physiology of a biofilm^52^ determining whether secreted Pel alters the degree of oxygenation in a pellicle environment would shed light on the mechanisms of pellicle formation that secreted Pel contributes to.

Colony biofilms wrinkle when exopolysaccharides such as the *B. subtilis eps* dependent exopolysaccharide, and *P. aeruginosa* Psl and Pel polysaccharides are produced^12,40^. In a colony biofilm of rod-shaped bacteria, cell growth and cell-cell interactions generate forces along the long axis of neighboring cells that are reduced by creating a central spoke wrinkle through cellular verticalization^49,50^. Continued cellular growth leads to “pinching-off” of verticalized cells and the cells being ejected. These ejected cells create the spoke wrinkles^49^. PelA’s glycoside hydrolase activity reduces cell adherence to surfaces. As cell to surface adhesion is known to influence biofilm morphology, the absence of spoke wrinkles in the PA14 *pelA*^E218A^ colony biofilm (Fig. 5b and Supplementary Fig. 4) suggests that the generation of secreted Pel may be necessary for cellular ejection^49^. The mechanism of how secreted Pel alters the forces involved in this process remains to be determined.

Colony biofilm surface hydrophobicity is conferred by both surface chemistry and wrinkling, where the latter forms air filled cavities that push against a liquid droplet^42^. The *eps-dependent* exopolysaccharide is the primary component conferring surface non-wettability to *B. subtilis* colony biofilms, as both wrinkles and the amphiphilic BslA protein layer are lost in a *B. subtilis eps* mutant strain^40,41^. The loss of secreted Pel results in an intermediate wetting phenotype (Fig. 5c). This suggests that secreted Pel plays a role in conferring non-wettability, altering the surface topology and potentially the surface chemistry. Both the deletion of *B. subtilis* BslA and absence of secreted Pel result in poor colony biofilm wrinkling with no spoke wrinkles and increased wettability, suggesting that the generation of secreted matrix components can play a role in colony biofilm wrinkle development which limits the penetration of antimicrobial liquids^42^.

Rheology has revealed that biofilm viscoelasticity is reflective of biofilm structure and composition^43^. Viscoelasticity, which describes how a material deforms from an applied stress, influences bacterial persistence during an infection because it impacts diffusion and therefore antimicrobial penetration^43^. Wrinkly colony biofilms are considered a two-layered system with a soft viscous liquid core encased in a stiff elastic skin that contributes to a colony biofilm’s ability to resist an applied stress^45^. The reduction in PA14 *pelA*^E218A^ colony biofilm stiffness observed (Fig. 5f) correlates with the reduced wrinkling (Fig. 5b), and like the loss of cohesion in a pellicle (Fig. 5a), could suggest altered Pel-protein/eDNA matrix interactions. The softer matrix would also suggest that diffusion within the biofilm has been affected, which is supported by the decreased Congo red binding observed for the PA14 *pelA*^E218A^ colony biofilm (Fig. 5b and Supplementary Fig. 4). Altered diffusion and loss of a potential secreted Pel-eDNA interaction has implications for the ability of the hydrolase mutant to resist antibiotic and mucolytic treatments (Fig. 7b).

We observed an increased host-killing rate in two different infection models using the PA14 *pelA*^E218A^ strain, suggesting that PelA hydrolase activity is involved in virulence modulation. How PelA hydrolase activity modulates virulence is unknown and could be related to changes in the virulence factors secreted, the cell surface, or the degree of Pel deacetylation. Notably, it has been established that oral infection of *Drosophila* by a non-biofilm-forming strain disseminates faster through the fly body than a biofilm-forming strain^48^. Our observation that PA14 *pelA*^E218A^ has the same killing kinetics as PA14 Δ*pelF* (Fig. 6c) suggests an altered host immune response. Immune responses in invertebrates such as *C. elegans* and *Drosophila* are commonly performed by epithelial cells^53–55^. These cells generate the reactive oxygen species (ROS) superoxide and hydrogen peroxide as a stress response to damage biological macromolecules^56,57^. Given that the exopolysaccharides alginate and cepacian produced by *P. aeruginosa* and members of the *Burkholderia cepacia* complex, respectively, in the CF lung have the capability to sequester ROS^58–60^, we hypothesize that the increased host killing exhibited by PA14 *pelA*^E218A^ could be due to a reduced ability of this mutant to sequester ROS in the matrix, suggesting secreted Pel may contribute to host immune response evasion (Fig. 7b).

In summary, we have established that the hydrolytic activity of PelA is responsible for the production of the low molecular weight secreted form of the Pel polymer. We found that PelA hydrolase activity contributes to biofilm biomechanics (Fig. 7a) and decreases *P. aeruginosa* PA14 virulence (Figs. 6a, c). We speculate that hydrolase activity may also modulate the chemical properties of the biofilm through ionic interactions (Fig. 7b). We have previously shown that deletion of *B. cereus pelA*_*h*_^*Bc*^ also results in a significant increase in adherent biofilm biomass^24^, suggesting that PelA_h_^Bc^ may also function to modify polymer length and biomechanics. Understanding the role of glycoside hydrolase activity in the biosynthesis of other exopolysaccharides will help establish whether the roles we have identified for PelA hydrolase activity are generalizable and why this activity has been evolutionary conserved. Finally, we propose that secreted biofilm matrix components be referred to as matrix associated to better reflect their active function. These studies have advanced not only our understanding of the matrixome but also the role of Pel, a widespread biofilm determinant.

## Methods

### Bacterial strains, plasmids, microbiological media, and growth conditions

Bacterial strains and plasmids used in this study are listed in Supplementary Table 1 and Supplementary Table 2, respectively. Lysogeny broth (LB) contained per liter of ultrapure water: 10 g tryptone, 10 g NaCl, and 5 g yeast extract. No-salt lysogeny broth (NSLB) was prepared as LB, excluding NaCl. Jensen’s medium contained per liter of ultrapure water: 5 g NaCl, 2.51 g K_2_HPO_4_, 15.56 g glutamic acid, 2.81 g L-valine, 1.32 g L-phenylalanine, 0.33 g/L MgSO_4_•7H_2_O, 21 mg CaCl_2_•2H_2_O, 1.1 mg FeSO_4_•7H_2_O, 2.4 mg ZnSO_4_•7H_2_O, and 1.25% (w/v) D-glucose. Vogel-Bonner minimal medium (VBMM) was prepared as a 10X concentrate containing per liter of ultrapure water: 2 g MgSO_4_•7H_2_O, 20 g citric acid, 100 g K_2_HPO_4_, and 35 g NH_4_HPO_4_ and was diluted to 1X as needed. To prepare solid medium, 1.5% (w/v) agar was added to LB, NSLB, or VBMM. *E. coli* and *P. aeruginosa* strains were grown at 37 ^°^C overnight with shaking at 200 RPM. Antibiotics were added to growth media, where appropriate. For *E. coli* transformant selection, 10 μg/mL gentamicin (GEN) and 50 μg/mL carbenicillin (CAR) was used for DH5α and XL10, respectively. For *P. aeruginosa*, 30 μg/mL GEN was used for plasmid maintenance of pERA4, and 60 μg/mL GEN was used for merodiploid selection.

### Standard molecular methods

All basic microbiological and molecular procedures were executed according to standard protocols^61^. Genomic DNA (gDNA) was isolated using Bio-Rad InstaGene matrix. Plasmid isolation and DNA gel extraction were performed using purification kits purchased from Qiagen or BioBasic. Restriction enzymes, T4 DNA ligase, Quick ligase, alkaline phosphatase, and Taq DNA polymerase were purchased from New England Biolabs or ThermoFisher Scientific. Phusion DNA polymerase and BP Clonase II were purchased from ThermoFisher Scientific. Primers used in this study were obtained from Sigma-Aldrich or Integrated DNA technologies (Supplementary Table 3). Site-directed mutagenesis of plasmids was completed using the Agilent QuikChange Lightning site-directed mutagenesis kit. Transformation of *P. aeruginosa* was performed using standard protocols for electroporation^62^. The miniTn7 constructs were introduced into the *P. aeruginosa* chromosome via electroporation with the helper plasmid pTNS2. Insertion of miniTn7 at the neutral attTn7 site adjacent to *P. aeruginosa glmS* was confirmed by PCR using the primers PTn7L, PTn7R, PglmS-up and PglmS-down (Supplementary Table 3)^63^. For genetic complementation of PA14 Δ*pelA*, the *pelA* nucleotide sequence from *P. aeruginosa* PA14 obtained from the Pseudomonas genome database^64^, a synthetic ribosome binding site nucleotide sequence (GAGGAGGATATTC), and the pPSV39 vector^65^ were submitted to BioBasic for their Gene Synthesis service which included cloning, ligation, and Sanger sequencing to generate the pERA4 plasmid (Supplementary Table 2). The pERA4 plasmid was introduced into PA14 Δ*pelA* by electroporation^62^. Positive transformants were selected on LB agar containing 30 μg/mL GEN. Sanger sequencing to validate the sequence of plasmids and chromosomal mutations (described below) was performed at The Center for Applied Genomics (TCAG) at The Hospital for Sick Children (SickKids), Toronto, Canada.

### Construction of allelic exchange vectors and P. aeruginosa PA14 mutants

In-frame, unmarked *pelF* and *pelA* gene deletions and the *pelA*^E218A^ point mutation were constructed in *P. aeruginosa* PA14 using established protocols for two-step allelic exchange^66^. Briefly, vectors containing in-frame Δ*pelA* and Δ*pelF* alleles were generated by cloning DNA fragments flanking the open reading frames (ORFs), which were subsequently joined by splicing-by-overlap extension PCR (SOE-PCR). In the case of Δ*pelA*, the SOE-PCR product was generated with primers containing EcoRI and HindIII sites (Supplementary Table 3) to enable restriction-based cloning. By contrast, the Δ*pelF* allele was generated with primers containing *attB1* and *attB2* sites (Supplementary Table 3) to enable cloning by Gateway technology. In both cases, SOE-PCR products were gel purified. Subsequently, the Δ*pelA* SOE-PCR product was digested with EcoRI and HindIII, and was ligated into similarly digested pEX18Gm^67^ (Supplementary Table 2). By contrast, Δ*pelF* SOE-PCR product was recombined with pDONRPEX18Gm^68^ (Supplementary Table 2) with BP Clonase II (Invitrogen). The allelic exchange vectors were transformed into *E. coli* DH5α and selected on LB agar containing 10 μg/mL GEN. Plasmids with the desired insert were identified by colony PCR using M13F and M13R primers (Supplementary Table 3). These clones were streaked on LB agar containing 10 μg/mL GEN, and the plasmids were subsequently purified and then verified by Sanger sequencing using the same M13F and M13R primers (Supplementary Table 3). This approach yielded pERA1 and pGBW2, which encoded the Δ*pelA* and Δ*pelF* alleles, respectively (Supplementary Table 2).

In the case of the allelic exchange vector for *pelA*^E218A^, DNA flanking the *pelA* E218 codon was cloned by PCR, gel purified, digested with EcoRI and HindIII, and ligated into pEX18Gm^67^. Clones bearing the desired insert were also identified by colony PCR and sequence verified as described above for the Δ*pelA* allele, yielding the pERA2 plasmid (Supplementary Table 2), which was subjected to site-directed mutagenesis (see below) to introduce the E218A mutation, resulting in the pERA3 plasmid (Supplementary Table 3).

In the case of the allelic exchange vector for Δ*pelA* in the Δ*wspF* Δ*psl* P_BAD_*pel* strain, which is a derivative of *P. aeruginosa* PAO1, the SOE-PCR product with an in-frame Δ*pelA* allele was generated as described above except that the 5′ fragment was cloned with primers targeting the interior of the *pelA* ORF (Supplementary Table 3, primer pair oJJH419/oJJH1415) so as not to interrupt the *P*_*BAD*_ promoter inserted upstream of *pelA*. By contrast, the 3′ fragment was cloned with primers designed with homology primarily to DNA flanking the *pelA* ORF (Supplementary Table 3, primer pair oJJH1416/oJJH1417). All other steps of generating the resulting allelic exchange vector, pJJH310, were carried out using Gateway cloning, colony PCR to identify the desired clone, and Sanger sequencing to verify the cloned insert as described above for the *P. aeruginosa* PA14 *ΔpelF* allele.

The pERA1, pERA3, pGBW2, or pJJH310 allelic-exchange plasmids were introduced into *P. aeruginosa* PA14 or PAO1 Δ*wspF* Δ*psl P*_*BAD*_*pel* strain via biparental mating with the donor strain *E. coli* SM10 (λ_pir_)^69^. Merodiploids were selected on VBMM agar containing 60 μg/mL GEN. Double crossover mutations were selected for by SacB-mediated counter selection on NSLB agar containing 15% (w/v) sucrose. Colonies were then screened for those that were GEN-sensitive and sucrose-resistant. Primers targeting the outside flanking regions of the *pelF* and *pelA* ORF were used to validate the mutations by colony PCR and Sanger sequencing (Supplementary Table 3). As required, mutations were further verified by whole genome re-sequencing.

### Genome re-sequencing and bioinformatics

gDNA was purified from *P. aeruginosa* strains PA14, JJH879, and GBW29. For *P. aeruginosa* JJH879, gDNA was sent to the Microbial Genome Sequencing (MiGS) Center, which was sequenced using their 200 Mbp Illumina®, paired-end sequencing package for the NextSeq 2000. By contrast, gDNA for all other strains was sequenced in-house. Here, sequencing libraries were prepared using the Nextera® XT DNA Library Preparation Kit (Illumina®) following the manufacturer’s directions. Sequencing libraries were multiplexed using the Nextera XT Index Kit (Illumina®). Where appropriate, DNA was purified using AMPure XP beads (Agencourt) and separation on a magnetic rack following the supplier’s protocols. Sequencing libraries were quantified using the DNA HS Assay kit (Invitrogen) for the Qubit 2.0 Fluorometer. The Agilent DNA 1000 kit and Bioanalyzer (Agilent®) were used to determine the library DNA size distributions following the manufacturer’s directions.

Paired-end sequencing was conducted using a MiSeq^TM^ System (Illumina®). All libraries were diluted to 4.0 nM in nuclease-free water and then pooled by aliquoting 5.0 µL of each library into a 1.5 mL Eppendorf tube. An aliquot of the pooled libraries was denatured by mixing 5.0 µL of the pooled libraries with 5.0 µL of 0.2 N NaOH. This mixture was centrifuged for 1 min at 750 × *g*, incubated at room temperature for 5 min, and then diluted with 990.0 µL of hybridization buffer and placed on ice. In parallel, the PhiX control library (10.0 nM, Illumina) was diluted to 4.0 nM using 10.0 mM Tris pH 8.5 and then denatured and diluted identically to the pooled libraries. Finally, 6.0 µL of the denatured PhiX control library was added to 594.0 µL of the denatured pooled libraries. The libraries were incubated at 96 °C for 2 min, cooled in a water bath, and then loaded into the MiSeq Reagent kit V2 (150 cycles, Illumina), which was run according to the manufacturer’s directions.

FASTQ files were analyzed using Geneious Prime v2022.1.1 Build 2022-03-15 11:43 (Dotmatics) in Java v11.0.14.1 + 1. Reads were trimmed on quality (minimum Q-score ≥30) and filtered by length (≥50 bp) using BBDuk v38.84. These reads were then mapped to the *P. aeruginosa* PA14 (NCBI accession NC_008463.1) or PAO1 (NCBI accession NC_002516.1) reference genomes, as appropriate, using the Geneious Mapper with medium-low sensitivity that was set to find structural variants, short insertions, and deletions of any size. Engineered deletions were then verified via visualization of the *pel* locus.

### Site-directed mutagenesis

Amino acid changes in PelA were introduced by making nucleotide substitutions in the *pelA* gene using the QuikChange Lightening Site-Directed Mutagenesis Kit (Agilient). Primers (Supplementary Table 3) were designed based on the parameters described by the manufacturer and were used in PCR to amplify pERA2 and pCAS4 containing the desired mutation. Subsequently, DpnI was added to digest the template plasmids. The PCR products of digested pERA2 were transformed into *E. coli* DH5α (Supplementary Table 1) and spread on LB agar containing 30 μg/mL GEN. The PCR products of the digested pCAS4 were transformed into *E. coli* XL10 (Supplementary Table 1) and spread on LB agar containing 50 μg/mL CAR and 10 μg/mL GEN. Plasmid sequences were confirmed via Sanger sequencing using the primers oER7-oER8 and oJDR33 to oJDR40 (Supplementary Table 3).

### Crystal violet assay for biofilm formation in microtiter plates

Overnight cultures of *P. aeruginosa* grown to stationary phase in NSLB supplemented with 30 μg/mL GEN, where appropriate, were diluted to a final OD_600nm_ of 0.005 in 1 mL of NSLB. Cultures containing PBADpel-derived strains were supplemented with 0.5% (w/v) L-(+)-arabinose to induce *pel* expression. Cultures containing strains expressing pPSV39 were supplemented with 30 μg/mL GEN for plasmid maintenance. 100 μL of the normalized cultures were added to the wells of a Corning CellBind 96-well microtiter plate (*n* = 5 for each strain examined) and incubated for 20 h at 25 °C statically. Non-adherent biomass was removed by washing the wells three times with water, and the remaining adherent biomass was stained with 150 μL of 0.1% (w/v) crystal violet for 10 min with agitation at room temperature. To remove excess stain, the wells were washed three times with water and the residual stain was solubilized by adding 200 μL of anhydrous ethanol to each well and left to incubate for 10 min with agitation at room temperature. 40 μL of the solubilized crystal violet solution was transferred to a plate containing 160 μL of anhydrous ethanol, and the absorbance was measured at 595 nm to quantify the biofilm biomass.

### Transmission electron microscopy

Single colonies were inoculated into 3 mL LBNS and grown overnight with shaking (200 rpm) at 37 °C. Overnight cultures were back-diluted (100 µL) into 25 mL of LBNS and grown with shaking (200 rpm) for 8 h at either 37 °C for P_BAD_*pel* strains or 25 °C for PA14 strains. Arabinose (0.5% (w/v)) was included in the media for the P_BAD_*pel* strains. A few drops of bacterial culture were applied to a grid (carbon film on 300 mesh copper grids; Electron Microscopy Sciences, Hatfield, PA), and the grid was allowed to absorb the cells for 2 min. The liquid was removed with filter paper, and the grid was rinsed by dipping it in five large drops of deionized water. The liquid was again removed with filter paper. Uranyl acetate (10 µL of 0.2% (w/v) uranyl acetate prepared in water) was applied to the grid for 90 s, and then the liquid was removed with filter paper. The grid was allowed to air dry. Transmission electron microscopy was performed on the Philips CM100 transmission electron microscope at The Biology Imaging Facility at the University of Washington.

### Viable cell counts

To obtain CFU counts from planktonic cultures, overnight cultures of *P. aeruginosa* grown to stationary phase in NSLB supplemented with 30 μg/mL GEN, where appropriate, were diluted to a final OD_600nm_ of 1 in 1 mL of phosphate-buffered saline (PBS). The normalized cultures were then serially diluted 1:10 in 500 μL PBS to a maximum dilution factor of 10^8^. 25 μL of cultures diluted 10^5^ to 10^8^ were spotted on LBNS agar supplemented with 0.5% (w/v) L-(+)-arabinose and 30 μg/ml GEN, where appropriate, and incubated for 48 h at 25 °C. CFUs and dilution factors were recorded from spots with countable colonies, averaged, and the final CFU/mL was calculated. To enumerate CFU counts from biofilms, cultures were grown as described above for the crystal violet microtiter plate assay. Biofilms were disrupted with 2 μM PelA_h_^Pa^ for 15 min with agitation at room temperature to release biofilm cells. The disrupted biofilms were diluted to a final OD_600nm_ of 1 in 50 μL of PBS. The subsequent diluting, plating, and growth was performed as described for CFU counts from planktonic cultures. CFU counts from planktonic cultures and biofilms were enumerated from three independent experiments (*n* = 2 spots with countable colonies per strain per experiment).

### Dot blots

PBADpel-derived strains were grown overnight at 37 °C to stationary phase in 1 mL Jensen’s media supplemented with 0.5% (w/v) L-(+)-arabinose to induce *pel* expression. Cultures were centrifuged at 16,000 x *g* for 2 min. The supernatant containing secreted Pel was set aside. Cell pellets were resuspended in 100 μL 0.5 M ethylenediaminetetraacetic acid (EDTA), pH 8.0, boiled for 20 min, and centrifuged at 16,000 x *g* for 10 min to harvest the supernatant containing cell-associated Pel. Secreted and cell-associated Pel were treated with proteinase K at a final concentration of 0.5 mg/mL and heated at 60 °C for 60 min, then 80 °C for 30 min in inactivate proteinase K.

Pel antisera^18^ was adsorbed by incubating 12.5 μL Pel antisera with 16.5 μL PA14 Δ*pelF* lysate, 16.5 μL PAO1 Δ*wspF* Δ*psl* Δ*pel* lysate, 16.5 μL PA14 Δ*pelF* culture supernatant ethanol precipitate, and 150 μL 5% (w/v) skim milk tris-buffered saline (10 mM Tris-HCl (pH 7.5) and 150 mM NaCl) with 0.5% (v/v) Tween 20 (TBS-T) at room temperature with rotation for 3 h. The adsorbed α-Pel was diluted 1:60 in 1% (w/v) milk TBS-T. 5 μL of secreted and cell-associated Pel were pipetted on a nitrocellulose membrane and left to air dry for 10 min. The membrane was blocked with 5% (w/v) skim milk TBS-T for 1 h at room temperature and incubated with adsorbed α-Pel overnight at 4 °C with shaking. The membrane was washed with TBS-T three times for 5 min each, incubated with goat α-rabbit HRP-conjugated secondary antibody (Bio-Rad) at a 1:2000 dilution in TBS-T for 45 min at room temperature with shaking, and washed again with TBS-T. All dot blots were developed using SuperSignal West Pico (Thermo Scientific) following the manufacturer’s instructions.

For WFL-HRP dot blots, the nitrocellulose membrane with 5 μL secreted and cell-associated Pel were prepared as described above. The membrane was blocked with 5% (w/v) bovine serum albumin (BSA) in TBS-T for 1 h at room temperature and incubated with 10 μg/mL of WFL-HRP (EY Laboratories) in 2% (w/v) BSA in TBS-T with 0.2 g/L CaCl_2_ overnight at room temperature with shaking. The membrane was washed with TBS twice for 5 min and once for 10 min, and developed as outlined above.

### Western blot sample preparation and analysis

PBAD*pel*-derived strains were grown overnight at 37 °C to stationary phase in 5 mL NSLB supplemented with 0.5% (w/v) L-(+)-arabinose to induce *pel* expression. Cultures were normalized to an OD_600_ of 1, centrifuged at 27,000 x *g* for 3 min to obtain the cell pellet. The pellet was resuspended in 50 μL 2X Laemmli buffer, boiled for 30 min, vortexed for 2 min, and analyzed using SDS-PAGE followed by Western blotting.

For Western blot analysis, a 0.2 μm PVDF membrane was soaked in methanol and then in Western transfer buffer (25 mM Tris-HCl, 150 mM glycine, and 20% (v/v) methanol). Protein was transferred from the SDS-PAGE gel to PVDF membrane by wet blotting (25 mV, 1.5 h). The membrane was blocked in 5% (w/v) skim milk TBS-T for 30 min at room temperature with gentle rocking, washed twice for 5 min each with TBS-T, and incubated with primary antibody in 1% (w/v) skim milk TBS-T at 4 °C overnight. P_BAD_*pel* derived strains were analyzed for protein levels using an α-PelA antibody at a dilution of 1:500^18^. α-PilF was used a loading control at a dilution of 1:1000 ^70^. The membrane was washed four times in TBS-T for 5 min each, and then incubated for 1 h with secondary antibody (1:2,000 dilution of Bio-Rad affinity purified goat anti-rabbit IgG conjugated to alkaline phosphatase) in 1% (w/v) skim milk TBS-T at room temperature. The membrane was then washed three times with TBS-T for 5 min each before development with 5-bromo-4-chloro-3- indolylphosphate–nitroblue tetrazolium chloride (BioShop ready-to-use BCIP/NBT solution). Developed blots were imaged using a Bio-Rad ChemiDoc imaging system. All blots were derived from the same experiment and processed in parallel.

### Confocal flow cell biofilm imaging

Overnight cultures of the P_BAD_*pel-*derived strains were diluted to OD_600nm_ = 0.01 in a defined glucose rich media (85 mM NaCl, 12 mM K_2_HPO_4_, 83 mM glutamic acid, 24 mM L-valine, 8 mM L-phenylalanine, 1.34 mM MgSO_4_, 0.143 mM CaCl_2_, 4 µM FeSO_4_, 8.4 µM ZnSO_4,_ pH 7.3) supplemented with 1% (w/v) arabinose and grown to exponential phase (OD_600nm_ = 0.5). Exponential phase cultures were diluted to OD_600nm_ = 0.01 in a defined minimal glucose media (85 mM NaCl, 12 mM K_2_HPO_4_, 15.1 mM (NH_4_)_2_SO_4_, 1.34 mM MgSO_4_, 0.143 mM CaCl_2_, 4 µM FeSO_4_, 8.4 µM ZnSO_4,_ pH 7.0) supplemented with 0.2% (w/v) arabinose. Diluted cultures were inoculated into flow cells, the flow cells were inverted, and cells were allowed to attach under static conditions for 1 h. Biofilms were cultivated under continuous flow (10 mL/h) for 96 h. Biofilms were stained for 30 min with FITC-labeled WFL lectin (100 μg/mL; Bioworld) for Pel, and Syto62 red fluorescent dye (5 μM; Thermo fisher Scientific) for biomass. After staining, biofilms were washed with media at 10 mL/h for 5 min and then visualized on a Zeiss LSM 800 scanning confocal laser microscope. Image analysis was performed using the Volocity software (Quorum Technologies Inc.).

### Flocculation assay

Overnight cultures of P_BAD_*pel* and PA14-derived strains were grown as indicated above for the crystal violet microtiter plate assay at 25 °C and 220 RPM. 1 mL aliquots of cultures were incubated in 1.5 mL Eppendorf tubes statically at room temperature for 60 min, and then photographed. The assay was performed in triplicate with one representative image shown.

### Standing pellicle assay

PA14 strains were grown to stationary phase in NSLB and then diluted to a final OD_600nm_ of 0.005 in 3 mL of NSLB in borosilicate glass tubes. The glass tubes were left to grow statically at 25 °C for 72 h. The glass tubes were then photographed for pellicle formation, and contents tipped into a petri dish (WillCo-dish glass bottom) and photographed again to identify the pellicle class. The assay was performed in triplicate, with one representative image shown.

### Congo red colony morphologies

PA14 strains were grown to stationary phase in NSLB and normalized to a final OD_600nm_ of 0.5 in 50 μL NSLB. Four μL of normalized cultures were spotted onto agar plates containing 10 g/liter tryptone, 10 g/liter agar, 40 μg/ml Congo red, and 15 μg/ml brilliant blue R. The plates were incubated at 25 °C for five days, and then photographed. The assay was performed in triplicate, with one representative image shown.

### Contact angle wettability

The wettability of colony biofilms was evaluated by measuring the static water contact angle using the sessile drop method^71^. Colony biofilms were grown as described above for Congo red colony morphologies for 6 d. Colony biofilms were carefully cut and transferred to clean microscope slides, and then placed on the stage of our custom-made contact angle system. A 10 µL drop of double distilled water was gently placed onto the center of each colony and imaged. The contact angle (θ) between the horizontal plane and the tangent to the drop at the point of contact with the surface was analyzed by ImageJ^72^ using the contact angle plugin (M. Brugnara, University of Trento, Trento, Italy 2010). Contact angles on colony biofilms were measured across four biological replicates and the average value and standard deviation were reported.

### Indentation rheology

Overnight cultures of *P. aeruginosa* strains were normalised to OD_600nm_ of 0.5 into NSLB. Sterile nitrocellulose filter membranes (25 mm, 0.45 μm pore size; Milliopore) were transferred onto NSLB media solidified with 1.5% agar (LANS). 100 μL of normalised culture was transferred onto filter membranes and spread evenly to cover the entire filter. Colony biofilms were grown at 25 °C for 7 d, transferring the biofilm-coated filters to a new LANS plate every 24 h. Uniaxial mechanical indentation of *P. aeruginosa* colony biofilms was performed on a TA Instruments Discovery Hybrid Rheometer-2 (DHR-2) with the Peltier plate connected to a heat exchanger (TA Instruments). Biofilms were analysed at 25 °C. The instrument was fitted with an 8 mm Smart-Swap sandblasted parallel plate geometry, with an approach rate of 1 μm/s. The Peltier plate was covered with a moist Kimwipe™ to prevent desiccation of the biofilm during analysis. TRIOS v5 (TA instruments) was used for data collection. The Young’s modulus (E) was determined using the force-displacement relationship previously described^44^:

$$E = \frac{{slope \cdot (1 - v^2)}}{{2r}}$$

where *slope* is the slope of the force-displacement curve (N/m), r is the radius of the probe (*r* = 0.004 m) and *ν* is the assumed Poisson’s ratio of a biofilm (*ν* = 0.5)^73^. The *slope* is calculated from the lower linear region of the force-displacement curve, which corresponded to 0–30% strain where R^2^ > 0.9. For indentation rheology measurements, four independent experiments (*n* = 2 biofilms per PA14 strain per experiment) were performed.

### C. elegans fast and slow killing survival assays

*C. elegans strain* N2 (Wild-type, Bristol strain) was obtained from the Caenorhabditis Genetics Center (CGC). Worms were maintained at 21 °C on nematode growth media (NGM) plates seeded with 10X OP50-1 *E. coli*. For preparation of 10X OP50-1, cultures were grown to saturation in LB at 37 °C for 16–18 h. *C. elegans* populations were synchronized by washing worms off plates with M9 solution and bleaching with sodium hypochlorite/1 M NaOH until the embryos of gravid adults were released into solution. Eggs were washed three times with M9, resuspended in 5 mL M9, and rotated at 21 °C for 18–24 h to allow embryos to hatch into L1s. For pelleting of live worms, animals were centrifuged in microcentrifuge tubes for 30 s at 1400 x *g*.

For the *C. elegans* infection assays, PA14-derived strains were grown in 3 mL LB overnight at 37 °C from a single bacterial colony. A volume of 20 μL of bacterial culture was used to seed 3.5 cm fast killing (peptone-glucose-sorbitol; 1% Bacto-Peptone, 1% NaCl, 1% glucose, 0.15 M sorbitol, and 1.7% Bacto-Agar) plates or slow killing plates (nematode growth medium; 3 g NaCl, 3.5 g bacto-peptone, 17 g bacto-agar, 5 mg/mL cholesterol dissolved in 1 mL ethanol, 1 mL 1 M CaCl_2_, 1 mL 1 M MgSO_4_, and 25 mL 1 M potassium phosphate buffer, pH 6.0). To prevent *C. elegans* pathogen avoidance behaviour^74^, bacterial culture was spread to ensure plates were fully covered with *P. aeruginosa*. Seeded plates were incubated for 24 h at either 25 °C or 37 °C for growth of bacterial lawns for the fast- and slow-killing assays, respectively. Plates were maintained for a further 24 h at room temperature (RT) prior to infection assays. To assay survival, >30 L4 worms were transferred to seeded fast or slow killing plates and incubated at 25 °C. For slow-killing assays, worms were moved onto fresh seeded plates every 24 h to ensure F1 progenies were not included in survival analyses. Survival was monitored over 24 or 72 h for fast or slow killing assays, respectively. Worms that failed to respond to pressure from a metal pick were considered non-viable. Worms that crawled off plates due to avoidance behaviours were excluded from analyses. P values for the fast killing assay were determined by one-tailed, unpaired Student’s t-test using Prism software (GraphPad Software Inc.) with Bonferroni correction for multiple-testing. *P* values for the slow-killing assay were determined using the log-rank Mantel-Cox test using Prism software. For the slow-killing assay, three independent experiments (*n* = 105–120 worms per PA14 strain per experiment) were performed. For the fast-killing assay, eight independent experiments (*n* = 35–40 worms per PA14 strain per experiment) were performed.

### P. aeruginosa *oral infection of* D. melanogaster

Mid-log cultures of PA14-derived strains grown in LB at 37 °C were normalized to an OD_600_ of 25 in 5% (v/v) sucrose. Wild-type Canton-S (CS) flies were maintained on standard cornmeal fly food, on a 12/12-h light/dark cycle, at 25 °C and 45–50% relative humidity unless otherwise specified. 0-2 d old males were collected under short CO_2_ exposure and placed on standard fly food at a density of 20 flies per vial. 1-3 d old flies were transferred to 1% agar vials and starved for 4 hours to ensure that flies fed on bacteria as soon as they were provided with them. After starvation, flies were transferred to experimental vials (Diamed, Polystyrene Narrow Vials, 32-116) containing 5 mL of solidified 5% (w/v) filter-sterilized sucrose containing 1% agar. A sterile filter (Fisher Brand; Thick, Cat N 05-714-4) was placed on the top of the solidified sucrose and 250 μL of bacterial culture resuspended in 5% (w/v) of sucrose was added to the filter about 30 min before the start of the experiment to allow the filter to completely soak in the bacterial culture. Control flies were maintained on filters soaked with 5% (w/v) sucrose without bacteria. Vials containing flies were maintained horizontally to prevent flies from getting stuck on wet filters. Experimental vials were kept at 23 °C for the duration of the experiment and monitored every 24–48 hours for survivorship until all flies on filters with bacteria died. The moisture level of all filters was checked frequently to confirm the presence of food and water for flies. Prism software (GraphPad Software Inc.) was used to generate the Kaplan–Meier survivorship curves. Statistica software package was used to perform log-rank Mantel-Cox tests for estimation of the survival rates. Six independent experiments (*n* = 20 flies per PA14 strain per replicate) were performed.

### Reporting summary

Further information on research design is available in the Nature Research Reporting Summary linked to this article.

## Supplementary information

Supplementary Material

Reporting Summary

## Data Availability

All data generated or analysed during this current study are included in this published article and its supporting information.
