# Supplementary material for: Glycoside hydrolase processing of the Pel polysaccharide alters biofilm biomechanics and Pseudomonas aeruginosa virulence
Source: NPJ Biofilms Microbiomes. 2023 Feb 2;9:7. doi: 10.1038/s41522-023-00375-7 (PMC9894940; doi:10.1038/s41522-023-00375-7)
Supplement: Supplementary file 1 — Supplementary Material [file 41522_2023_375_MOESM1_ESM.pdf]

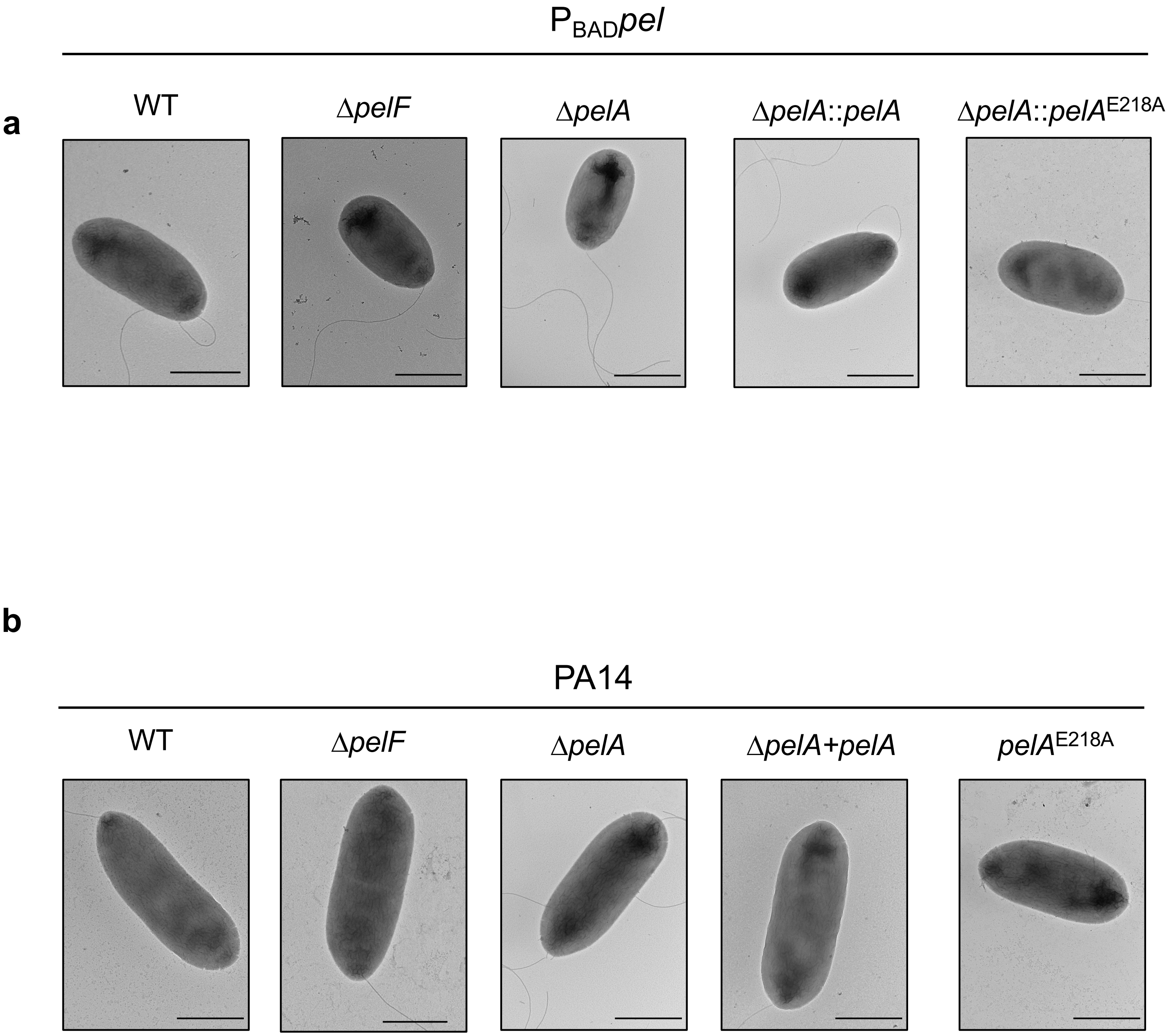

Supplementary Figure 1: Transmission electron microscopy micrographs of P_{BAD} *pel* and PA14 strains show normal cell morphologies. Related to Figure 2. (A) P_{BAD} *pel* (B) PA14 strains. Scale bars = 1 μm. P_{BAD} *pel*, PAO1 Δ*wspF* Δ*psl* P_{BAD} *pel.*

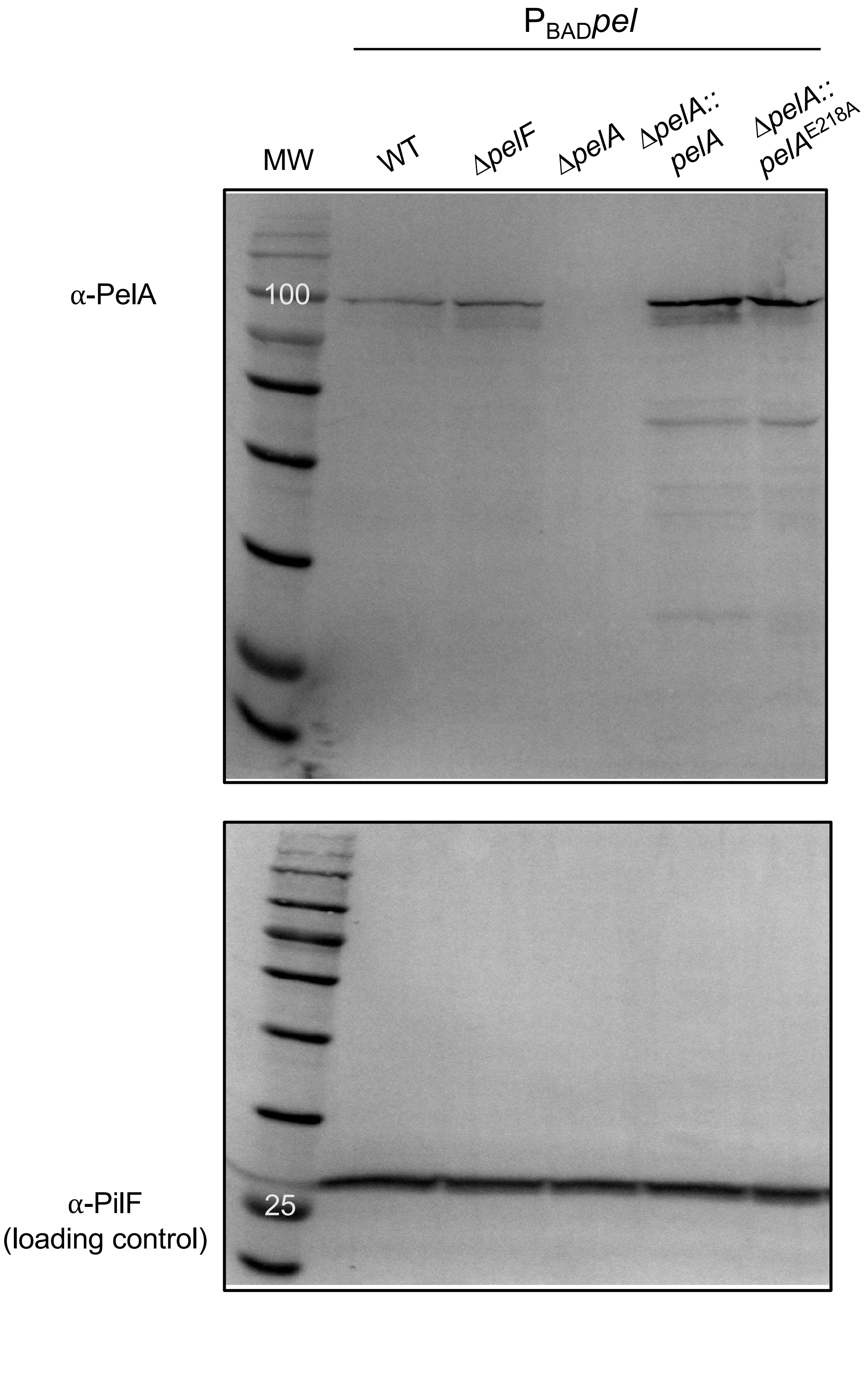

Supplementary Figure 2: Western blot probing for PelA expression levels in indicated P_{BAD} *pel* derived strains. Related to Figure 4. The molecular weight is indicated in kilodaltons in the MW lane on top of the marker band. The molecular weights of proteins are as follows: PelA, 101.1 kDa and PilF, 28.5 kDa. P_{BAD} *pel*, PAO1 Δ*wspF* Δ*psl* P_{BAD} *pel*, MW, molecular weight marker.

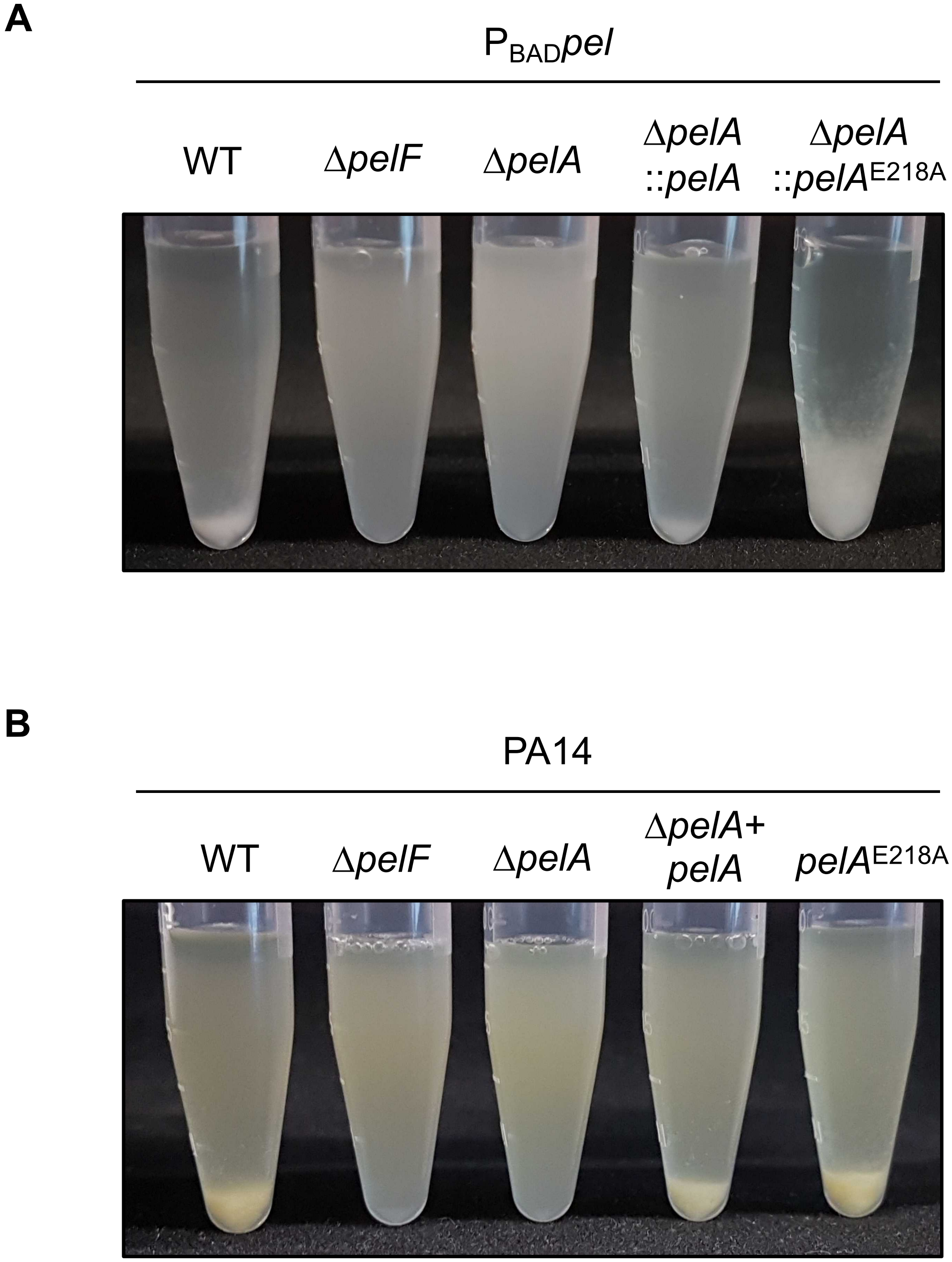

Supplementary Figure 3: PelA hydrolase mutant is more flocculent than wild type. Related to Figure 4. Overnight cultures of indicated strains grown to mid-log phase at 25°C and 220 RPM, transferred to an Eppendorf tube, left to sit statically for 60 min to sediment. P_{BAD} *pel*, PAO1 Δ*wspF* Δ*psl* P_{BAD} *pel.*

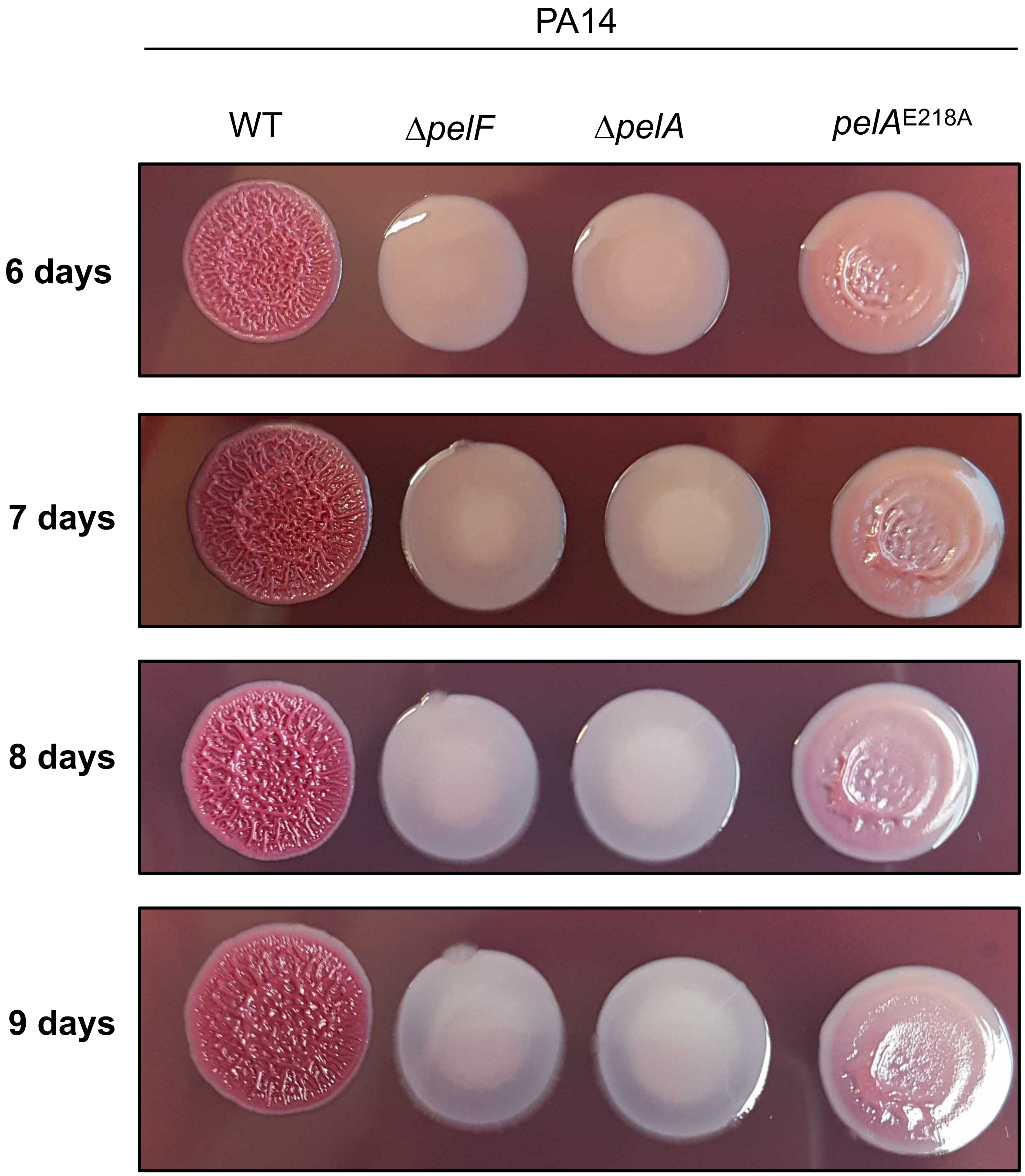

Supplementary Figure 4: Congo red colony morphologies of PA14 strains over several days. Related to Figure 5.

Supplementary Table 1: Bacterial strains used in this study.

Strains	Genotype or relevant characteristics[1]	Source
Pseudomonas aeruginosa		
PAO1	Wild type strain from Colin Manoil, genome re-sequenced	(1)
PAO1 Δ*wspF* Δ*psl* P_{BAD}*pel*	In-frame deletion of *wspF*, polar deletion of *pslBCD*, *araC*-P_{BAD} inserted upstream of *pelABCDEFG*	(2)
JJH879	PAO1 Δ*wspF* Δ*psl* P_{BAD}*pel* Δ*pelA*	This study
JJH885	PAO1 Δ*wspF* Δ*psl* P_{BAD}*pel* Δ*pelF*	(3)
LSM30	Gen^r, JJH879 *attTn7*::miniTn7T2.1-Gm-GW::*araC*-P_{BAD}::*pelA*	(4)
JDR27	Gen^r, JJH879 *attTn7*::miniTn7T2.1-Gm-GW::*araC*-P_{BAD}::*pelA*^{E218A}	This study
JDR82	Gen^r, JJH879 *attTn7*::miniTn7T-Gm	This study
PA14	Wild type strain	(2)
GBW29	PA14 Δ*pelF*	This study
ER1	PA14 Δ*pelA*	This study
ER2	PA14 *pelA*^{E218A}	This study
gacA SW7.4	Kan^r, PA14 *gacA::aphA-3*, non-polar insertion interrupting *gacA*	(5)
Escherichia coli		
DH5α	*F⁻ Φ80lacZΔM15* Δ*(lacZYA-arg*F*) U169 rec*A1 *end*A1 *hsd*R17 *(rK–, mK+) pho*A *sup*E44 *λ– thi-1 gyr*A96 *rel*A1	Invitrogen
SM10 (λ_{pir})	Kan^R, Tet^R. *thi thr leu tonA lacY supE recA::RP4-2-Tc::Mu* K_m λ_{pir}	(6)
ccdB Survival 2™ T1R	Str^R, strain for cloning plasmids with Gateway donor sites	Invitrogen
XL1-Gold	Strain for cloning	Agilent Technologies

[1]Abbreviations for antibiotic selection: Gen, gentamicin; Kan, kanamycin; Str, streptomycin; Tet, tetracycline.

Supplementary Table 2: Plasmids used in this study.

Plasmids	Genotype or relevant characteristics[1]	Source
**Allelic exchange vectors**		
pEX18Gm	Gen^R, suicide vector for allelic exchange in *P. aeruginosa*, SacB, lacZα	(6)
pDONRPEX18Gm	Gen^R, Cam^R, pEX18Gm with a Gateway® donor site	(1)
pERA1	Gen^R, pEX18Gm with *P. aeruginosa* PA14 Δ*pelA*	This study
pERA2	Gen^R, pEX18Gm with a fragment encoding a portion of *P. aeruginosa* PA14 *pelA* (402 bp upstream and 477 bp downstream of E218)	This study
pERA3	Gen^R, pERA2 with $pelA^{E218A}$	This study
pGBW2	Gen^R, pDONRPEX18Gm with *P. aeruginosa* PA14 Δ*pelF*	This study
pJJH310	Gen^R, pDONRPEX18Gm with *P. aeruginosa* PAO1 Δ*pelA*	This study
**Complementation analysis**		
pUC18-mini-Tn7T2.1-Gm-GW	Amp^R, Gen^R, Cam^R, miniTn7 with transcriptional terminators flanking the Tn7 transposon; Gateway destination vector	(7)
pUC18T-miniTn7T-Gm	Amp^R, Gen^R, miniTn7 vector	(8)
pTNS2	Amp^R, helper plasmid encoding *tnsABCD*	(8)
pCAS4	Gen^R, Amp^R, pUC18-miniTn7T2.1-Gm-GW with *araC*- P_{BAD}::*pelA* in the Gateway cloning site	(4)
pJDR40	Gen^R, Amp^R, pCAS4 with P_{BAD}::$pelA^{E218A}$	This study
pPSV39	Gen^R, expression vector with *lacI* and *lacUV5* promoter derived from pPSV35	(9)
pERA4	Gen^R, RBS and full-length *pelA* from *P. aeruginosa* PA14 in pPSV39	This study

[1]Abbreviations for antibiotic selection: Amp, ampicillin; Cam, chloramphenicol; Kan, kanamycin; Gen, gentamicin.

Supplementary Table 3: Primers used in this study.

Primer name or identifier	Sequence[1]
**Sequencing primers**	
oER1_pelAF-SEQ	CGG CGA AAA CCT GCG GTG TT
oER2_pelAR-SEQ	CGG CCA GTT CGG CAA AGG T
oJJH1472_pelFF01-SEQ	CTA CTC GAT GCT CGA CCA GAA G
oJJH1473_pelFR01-SEQ	GAT GCG TTT GTA GGC CTT CAT TC
M13F(-20)	GT AAA ACG ACG GCC AGT
M13R(-27)	CAG GAA ACA GCT ATG AC
oJDR33_*araC-P$_{ara}$BAD*-500F01-SEQ	GGT GCG CTT CAT CCG GGC G
oJDR34_*araC-P$_{ara}$BAD*-1000F01-SEQ	GCA TTC TGT AAC AAA GCG G
oJDR35_PelA-308R01-SEQ	CGA TGG CGG CGG CGT CG
oJDR36_PelA-770R01-SEQ	GCG GCA GGT AGT CGA TGG CG
oJDR37_PelA-1308R01-SEQ	GCC GGC AGG TCG CGG ATC CG
oJDR38_PelA-1808R01-SEQ	CTG CCA GAA GAA CGG ATG G
oJDR39_PelA-2308R01-SEQ	GGT GCA TGG TGC GGA TCG
oJDR40_PelA-2808R01-SEQ	CAT CGC GCA CCT GCT CCA TC
oJJH373_PglmS-down	GCA CAT CGG CGA CGT GCT CTC
oJJH374_PglmS-up	CTG TGC GAC TGC TGG AGC TGA
oJJH371_PTn7R	CAC AGC ATA ACT GGA CTG ATT TC
oJJH372_Tn7L	ATT AGC TTA CGA CGC TAC ACC C
**Construction of allelic exchange vectors**	
oER3_pelAupF-EcoRI	GGG **GAA TTC** GCC TGG AAA GGA CAA CCT GA
oER4_pelAupR	*CGC GCA CCT GCT CCA TCG GCA A* TCC TTT CTT GCT GAA CCG CAT
oER5_pelAdownF	TTG CCG ATG GAG CAG GTG CGC G
oER6_pelAdownR-HindIII	GGG **AAG CTT** TTC GAG CAT GGC GTC GTT CA

oJJH1419_pelAupF03-GWB1	GGG G**AC AAG TTT GTA CAA AAA AGC AGG CTA** CGA GCT TTC CCA GTT CGA CTG
oJJH1415_pelAupR01	*CCA TCG CGC ACC TGC TCC ATC GG*G GCA GTG CCG GCA CAA AG
oJJH1416_pelAdownF01	CCG ATG GAG CAG GTG CG
oJJH1417_pelAdownR01-GWB2	GGG G**AC CAC TTT GTA CAA GAA AGC TGG GTA** CAA GGA TGT CCA GTT CCA CC
oJJH1435_pelFupF01-GWB1	GGG G**AC AAG TTT GTA CAA AAA AGC AGG CTA C**GC TGG TAC TGG GAA CTG GC
oJJH1436_pelFupR01	*GCA ATC TCC GTG GCT TCG CGG TAC AG*C GGA GCG GTG TGT TCG GTC
oJJH1437_pelFdownF01	CTG TAC CGC GAA GCC ACG G
oJJH1438_pelFdownR01-GWB2	GGG G**AC CAC TTT GTA CAA GAA AGC TGG GTA** CAG GGT CGC CAG CAA TAT CG
oER7_pelA-E218A-upF-EcoRI	GGG **GAA TTC** GGG TAT CTG AAA GAG CAG GGC
oER8_pelA-E218A-downR-HindIII	GGG **AAG CTT** GAT CCA GTT GTC GAA GGC GTC
**Site Directed Mutagenesis**	
oJJH1505_pelA-E218A-F01	GCG GTG GCC GTG GcG TCG ATC CAT GCC
oJJH1506_pelA-E218A-R01	GGC ATG GAT CGA CgC CAC GGC CAC CGC
oER9_pelA-E218A-F	TCG GCG GTG GCC GTG GcG TCG ATC CAT GCC GGT
oER10_pelA-E218A-R	ACC GGC ATG GAT CGA CgC CAC GGC CAC CGC CGA

[1]Restriction and attachment sites are bolded; regions complementary to the target amplicon are underlined; regions of reverse complementarity (to facilitate splicing) are italicized; lowercase letters denote a nucleotide substitution.

Supplementary references

1. **Harrison JJ**, **Almblad H**, **Irie Y**, **Wolter DJ**, **Eggleston HC**, **Randall TE**, **Kitzman JO**, **Stackhouse B**, **Emerson JC**, **Mcnamara S**, **Larsen TJ**, **Shendure J**, **Hoffman LR**, **Wozniak DJ**, **Parsek MR**. 2020. Elevated exopolysaccharide levels in Pseudomonas aeruginosa flagellar mutants have implications for biofilm growth and chronic infections. PLoS Genet **16**:1–22.

2. **Colvin KM**, **Alnabelseya N**, **Baker P**, **Whitney JC**, **Howell PL**, **Parsek MR**. 2013. PelA Deacetylase Activity Is Required for Pel Polysaccharide Synthesis in Pseudomonas aeruginosa. J Bacteriol **195**:2329–2339.

3. **Whitfield GB**, **Marmont LS**, **Ostaszewski A**, **Rich JD**, **Whitney JC**, **Parsek MR**, **Harrison JJ**, **Howell PL**. 2020. Pel Polysaccharide Biosynthesis Requires an Inner Membrane Complex Comprised of PelD, PelE, PelF, and PelG. J Bacteriol **202**:471–18.

4. **Marmont LS**, **Whitfield GB**, **Rich JD**, **Yip P**, **Giesbrecht LB**, **Stremick CA**, **Whitney JC**, **Parsek MR**, **Harrison JJ**, **Howell PL**. 2017. PelA and PelB form a modification and secretion complex essential for Pel polysaccharide-dependent biofilm formation in Pseudomonas aeruginosa. J Biol Chem jbc.M117.812842–24.

5. **Rahme LG**, **Tan M-W**, **Le L**, **Wong SM**, **Tompkins RG**, **Calderwood SB**, **Ausubel FM**. 1997. Use of model plant hosts to identify *Pseudomonas aeruginosa* virulence factors. Proc Natl Acad Sci USA **94**:13245–13250.

6. **Hoang TT**, **Karkhoff-Schweizer RR**, **Kutchma AJ**, **Schweizer HP**. 1998. A broad-host-range Flp-FRT recombination system for site-specific excision of chromosomally-located DNA sequences: application for isolation of unmarked Pseudomonas aeruginosa mutants. Gene **212**:77–86.

7. **Zhao K**, **Tseng BS**, **Beckerman B**, **Jin F**, **Gibiansky ML**, **Harrison JJ**, **Luijten E**, **Parsek MR**, **Wong GCL**. 2013. Psl trails guide exploration and microcolony formation in Pseudomonas aeruginosa biofilms. Nature **497**:388–392.

8. **Choi K-H**, **Schweizer HP**. 2006. mini-Tn7 insertion in bacteria with single attTn7 sites: example Pseudomonas aeruginosa. Nat Protoc **1**:153–161.

9. **Rietsch A**, **Vallet-Gely I**, **Dove SL**, **Mekalanos JJ**. 2005. ExsE, a secreted regulator of type III secretion genes in Pseudomonas aeruginosa. Proc Natl Acad Sci USA **22**:8006–8011.
