# Supplementary material for: Glycoside hydrolase processing of the Pel polysaccharide alters biofilm biomechanics and Pseudomonas aeruginosa virulence
Source: NPJ Biofilms Microbiomes. 2023 Feb 2;9:7. doi: 10.1038/s41522-023-00375-7 (PMC9894940; doi:10.1038/s41522-023-00375-7)
Supplement: Supplementary file 2 — Reporting Summary [file 41522_2023_375_MOESM2_ESM.pdf]

Corresponding author(s): P. Lynne Howell
Last updated by author(s): Oct 13, 2022

Reporting Summary

Nature Portfolio wishes to improve the reproducibility of the work that we publish. This form provides structure for consistency and transparency in reporting. For further information on Nature Portfolio policies, see our Editorial Policies and the Editorial Policy Checklist.

Statistics

For all statistical analyses, confirm that the following items are present in the figure legend, table legend, main text, or Methods section.

n/a	Confirmed	
☐	☒	The exact sample size (*n*) for each experimental group/condition, given as a discrete number and unit of measurement
☐	☒	A statement on whether measurements were taken from distinct samples or whether the same sample was measured repeatedly
☐	☒	The statistical test(s) used AND whether they are one- or two-sided<br>*Only common tests should be described solely by name; describe more complex techniques in the Methods section.*
☒	☐	A description of all covariates tested
☒	☐	A description of any assumptions or corrections, such as tests of normality and adjustment for multiple comparisons
☐	☒	A full description of the statistical parameters including central tendency (e.g. means) or other basic estimates (e.g. regression coefficient) AND variation (e.g. standard deviation) or associated estimates of uncertainty (e.g. confidence intervals)
☐	☒	For null hypothesis testing, the test statistic (e.g. *F*, *t*, *r*) with confidence intervals, effect sizes, degrees of freedom and *P* value noted<br>*Give P values as exact values whenever suitable.*
☒	☐	For Bayesian analysis, information on the choice of priors and Markov chain Monte Carlo settings
☒	☐	For hierarchical and complex designs, identification of the appropriate level for tests and full reporting of outcomes
☒	☐	Estimates of effect sizes (e.g. Cohen's *d*, Pearson's *r*), indicating how they were calculated

Our web collection on statistics for biologists contains articles on many of the points above.

Software and code

Policy information about availability of computer code

Data collection	Microsoft Excel version 16.54.
Data analysis	Graphpad Prism version 9 and Statistica Software version 14.

For manuscripts utilizing custom algorithms or software that are central to the research but not yet described in published literature, software must be made available to editors and reviewers. We strongly encourage code deposition in a community repository (e.g. GitHub). See the Nature Portfolio guidelines for submitting code & software for further information.

Data

Policy information about availability of data

All manuscripts must include a data availability statement. This statement should provide the following information, where applicable:

- Accession codes, unique identifiers, or web links for publicly available datasets
- A description of any restrictions on data availability
- For clinical datasets or third party data, please ensure that the statement adheres to our policy

All data generated or analysed during this current study are included in this published article and its supporting information

Human research participants

Policy information about studies involving human research participants and Sex and Gender in Research.

Reporting on sex and gender	N/A
Population characteristics	N/A
Recruitment	N/A
Ethics oversight	N/A

Note that full information on the approval of the study protocol must also be provided in the manuscript.

Field-specific reporting

Please select the one below that is the best fit for your research. If you are not sure, read the appropriate sections before making your selection.

☒ Life sciences ☐ Behavioural & social sciences ☐ Ecological, evolutionary & environmental sciences

For a reference copy of the document with all sections, see nature.com/documents/nr-reporting-summary-flat.pdf

Life sciences study design

All studies must disclose on these points even when the disclosure is negative.

Sample size	Sample size was determined using both technical and biological replicates, stated in the methods for each experiment. These sample sizes are sufficient as we ensured to have 3 or more total replicates to increase statistical power, and referred to the relevant literature to guide our sample size choices.
Data exclusions	No data was excluded.
Replication	To verify reproducibility of the experimental findings, we ensured to have both technical and biological replicates. We confirm all replication attempts were successful.
Randomization	Strains used in our study were allocated by the strain type i.e. the wild type and each mutant strain were grouped individually. Covariates were not relevant to control for our study as we found no confounding variables in our data.
Blinding	The investigators responsible for our infection model data using C. elegans and Drosophila were blinded to group allocation during data collection and analysis. Blinding was not performed for other experiments due to the nature of the experimental set up and the experiment being performed by a sole investigator.

Reporting for specific materials, systems and methods

We require information from authors about some types of materials, experimental systems and methods used in many studies. Here, indicate whether each material, system or method listed is relevant to your study. If you are not sure if a list item applies to your research, read the appropriate section before selecting a response.

Materials & experimental systems

n/a	Involved in the study	
☐	☒	Antibodies
☒	☐	Eukaryotic cell lines
☒	☐	Palaeontology and archaeology
☐	☒	Animals and other organisms
☒	☐	Clinical data
☒	☐	Dual use research of concern

Methods

n/a	Involved in the study	
☒	☐	ChIP-seq
☒	☐	Flow cytometry
☒	☐	MRI-based neuroimaging

Antibodies

Antibodies used	Two antibodies were used in this study. A polyclonal Pel antibody that cannot be commercially purchased. The production of this antibody is found in PMID: 26311845 and was provided to us by the lab of the corresponding author. A polyclonal PilF antibody was

	used as a loading control, and also cannot be commercially purchased. We previously published the generation of this antibody in PMID: 18776008.
Validation	The validation of the Pel antibody is shown in PMID: 26311845, in our work using the relevant positive and negative controls, and its application is to detect the Pel exopolysaccharide. The validation of the PilF antibody is shown in PMID: 18776008 and its application is to detect the PilF protein which is involved in localization of the Type IV pilus. We used this antibody in our work as a loading control specifically, as Pseudomonas aeruginosa contains PilF.

Animals and other research organisms

Policy information about studies involving animals; ARRIVE guidelines recommended for reporting animal research, and Sex and Gender in Research

Laboratory animals	This study did not involve laboratory animals.
Wild animals	This study did not involve wild animals.
Reporting on sex	Our findings using Drosophila melanogaster applied to only one sex, male. Sex was considered in the study design, which was previously developed in PMID: 21998591 because we were examining survival and needed to account for the potential of female flies being pregnant and confounding the data. The flies were assigned sex by standard visual inspection under a microscope. No data was disaggregated for sex because we only used male flies. Sex-based analyses were not performed because we only used male flies.
Field-collected samples	This study does not contain samples collected from the field.
Ethics oversight	No ethical approval or guidance was required because we used nematodes and insects in our study, not animals.

Note that full information on the approval of the study protocol must also be provided in the manuscript.
